# The root‐knot nematode effector MiEFF12 targets the host ER quality control system to suppress immune responses and allow parasitism

**DOI:** 10.1111/mpp.13491

**Published:** 2024-07-04

**Authors:** Salomé Soulé, Kaiwei Huang, Karine Mulet, Joffrey Mejias, Jérémie Bazin, Nhat My Truong, Junior Lusu Kika, Stéphanie Jaubert, Pierre Abad, Jianlong Zhao, Bruno Favery, Michaël Quentin

**Affiliations:** ^1^ INRAE‐Université Côte d'Azur‐CNRS, UMR Institut Sophia Agrobiotech Sophia Antipolis France; ^2^ State Key Laboratory of Vegetable Biobreeding, Institute of Vegetables and Flowers Chinese Academy of Agricultural Sciences Beijing China; ^3^ Institute of Plant Sciences Paris‐Saclay (IPS2) CNRS, INRAE, Université Paris Saclay – Evry, Université de Paris Gif sur Yvette France; ^4^ Present address: CIRAD, UMR PHIM Montpellier France; ^5^ Present address: Vietnamese‐German Center for Medical Research 108 Military Central Hospital Ha Noi Vietnam.

**Keywords:** effector, endoplasmic reticulum (ER), ER quality control, *Meloidogyne incognita*, *Nicotiana benthamiana*, *Solanum lycopersicum*

## Abstract

Root‐knot nematodes (RKNs) are microscopic parasitic worms able to infest the roots of thousands of plant species, causing massive crop yield losses worldwide. They evade the plant's immune system and manipulate plant cell physiology and metabolism to transform a few root cells into giant cells, which serve as feeding sites for the nematode. RKN parasitism is facilitated by the secretion in planta of effector molecules, mostly proteins that hijack host cellular processes. We describe here a conserved RKN‐specific effector, effector 12 (EFF12), that is synthesized exclusively in the oesophageal glands of the nematode, and we demonstrate its function in parasitism. In the plant, MiEFF12 localizes to the endoplasmic reticulum (ER). A combination of RNA‐sequencing analysis and immunity‐suppression bioassays revealed the contribution of MiEFF12 to the modulation of host immunity. Yeast two‐hybrid, split luciferase and co‐immunoprecipitation approaches identified an essential component of the ER quality control system, the *Solanum lycopersicum* plant bap‐like (PBL), and basic leucine zipper 60 (BZIP60) proteins as host targets of MiEFF12. Finally, silencing the *PBL* genes in *Nicotiana benthamiana* decreased susceptibility to *Meloidogyne incognita* infection. Our results suggest that EFF12 manipulates PBL function to modify plant immune responses to allow parasitism.

## Graphical abstract

The *Meloidogyne incognita* effector MiEFF12 targets endoplasmic reticulum‐resident proteins and corrupts their function in the host immune response to promote parasitism.

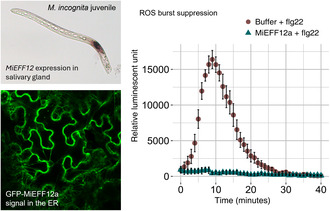

## 1 INTRODUCTION

Root‐knot nematodes (*Meloidogyne* spp., RKNs) are extremely polyphagous plant pathogens responsible for huge losses in agriculture (Jones et al., 2013). These obligate biotrophic root parasites manipulate plant functions to induce a permanent feeding structure. Following root penetration, the RKN second‐stage juveniles (J2s) migrate between the cells to reach the vascular cylinder, where they induce the redifferentiation of five to seven selected vascular parenchyma cells into hypertrophied multinucleate feeding cells or “giant cells”. These giant cells are the sole source of nutrients for the nematode and are essential for RKN development and reproduction. Concomitantly, the surrounding cells, xylem and phloem proliferate, inducing typical root deformations known as galls or root knots (Favery et al., 2020; Rutter et al., 2022). After successive moults, the adult female RKN lays eggs on the root surface. The success of RKNs as parasites depends on their ability to hijack essential host cell components to induce and maintain a functioning feeding site. The formation of this feeding site is mediated by the secretion into the host of effector proteins essential for RKN parasitism. These effectors hijack host cell processes, including those involved in immune responses, thereby facilitating successful parasitism (Rutter et al., 2022; Vieira & Gleason, 2019).

Plants can detect RKN infestation, which, like infections with other pathogens, triggers immune responses (Kaloshian & Teixeira, 2019; Sato et al., 2019; Siddique et al., 2022). Plants specifically recognize RKN pathogen‐associated molecular patterns (PAMPs) or damage‐associated molecular patterns (DAMPs) released by the RKNs during host invasion, via plasma membrane‐associated pattern‐recognition receptors (PRRs), which initiate an immune response known as pattern‐triggered immunity (PTI) (Goode & Mitchum, 2022; Huang et al., 2023; Sato et al., 2019; Siddique et al., 2022). PTI enables the plant to respond to nematode attack by producing reactive oxygen species (ROS), antimicrobial pathogenesis‐related proteins (PR) and metabolites, and by reinforcing their cell walls (Goode & Mitchum, 2022; Sato et al., 2019). In response, pathogens have evolved effector proteins that they secrete into the host to suppress PTI (Jones & Dangl, 2006). Plants have acquired resistance genes enabling them to recognize such effectors specifically, leading to the initiation of effector‐triggered immunity (ETI), which results in localized cell death through the hypersensitive response (HR) (Jones & Dangl, 2006; Sato et al., 2019). More than 20 RKN effectors have been reported to be involved in suppression of the PTI or ETI (Rutter et al., 2022). These effectors include MjShKT, which has a *Stichodactyla* toxin (ShKT) domain and is secreted by *Meloidogyne javanica* (Kumar et al., 2023). MjShKT has been implicated in the suppression of the ETI and is the only RKN effector known to target the host‐plant endoplasmic reticulum (ER) (Kumar et al., 2023).

The ER is a highly dynamic organelle consisting of a complex network of cisternae and tubules (Kriechbaumer & Brandizzi, 2020). It is physically connected to the nucleus, the plasma membrane, plastids and mitochondria, and is continuous between cells, via the plasmodesmata (Kriechbaumer & Brandizzi, 2020; Michaud & Jouhet, 2019). The ER, thus, participates in intracellular and intercellular communications. It is responsible for the biosynthesis and quality control of ER‐resident proteins and proteins destined for transportation to the vacuole, plasma membrane or apoplast (Kriechbaumer & Brandizzi, 2020). It is involved in lipid biosynthesis and storage (Kanehara et al., 2022) and is an important organelle for the storage of calcium (Ca^2+^), an instrumental intracellular messenger (Costa et al., 2018) involved in plant immune responses (Köster et al., 2022). The ER can, therefore, respond and adapt to the biosynthetic requirements imposed on plant cells during plant growth and by environmental stress (Brandizzi, 2021). The ER quality control system (ERQC) identifies misfolded proteins and directs them to the ER‐associated degradation (ERAD) machinery (Strasser, 2018). In the absence of such control, the accumulation of unfolded proteins within the ER would trigger ER stress, inducing the unfolded protein response (UPR), leading to the production of proteins of the ERAD pathway and of chaperones responsible for protein refolding, to restore ER homeostasis (Liu & Howell, 2016). If prolonged, the UPR eventually leads to plant cell death (Liu & Howell, 2016).

The plant ER and ERQC system components are targeted by several plant pathogen effectors (reviewed by Breeze et al., 2023; Jing & Wang, 2020). For example, proteins acting downstream from ER stress sensors and responsible for inducing the UPR, such as the BZIP60 and NAC transcription factors, have been shown to be targeted by pathogen effectors (Breeze et al., 2023; Jing & Wang, 2020). Similarly, effectors secreted by the oomycete *Phytophthora sojae* interact with ER‐resident chaperones, such as plant‐binding immunoglobulin proteins (BiPs), to suppress host immune responses (Jing et al., 2016). Consistently, plants with an impaired ability to sense ER stress or to trigger the UPR are generally more susceptible to pathogens (McLellan et al., 2013; Moreno et al., 2012; Zhang et al., 2015).

We describe here EFF12, an RKN‐specific effector expressed in the dorsal oesophageal glands and conserved in different *Meloidogyne* species. We show that MiEFF12 is involved in *Meloidogyne incognita* parasitism and associates with the host cell ER. A combination of transcriptomic and molecular analyses indicated that MiEFF12 was involved in suppressing host immune responses. We show that MiEFF12 interacts with the ER‐resident plant bap‐like (PBL), orthologues of the human B‐cell receptor‐associated protein 31 (BAP31 or BCAP31) and the basic leucine zipper 60 (BZIP60) proteins, two known components of ERQC systems. The silencing of *PBL* genes in *Nicotiana benthamiana* decreased susceptibility to *M. incognita*, suggesting a role for this protein in plant defence responses directed against RKNs.

## 2 RESULTS

### 2.1 EFF12 is an RKN‐specific effector required for parasitism

**FIGURE 1:**
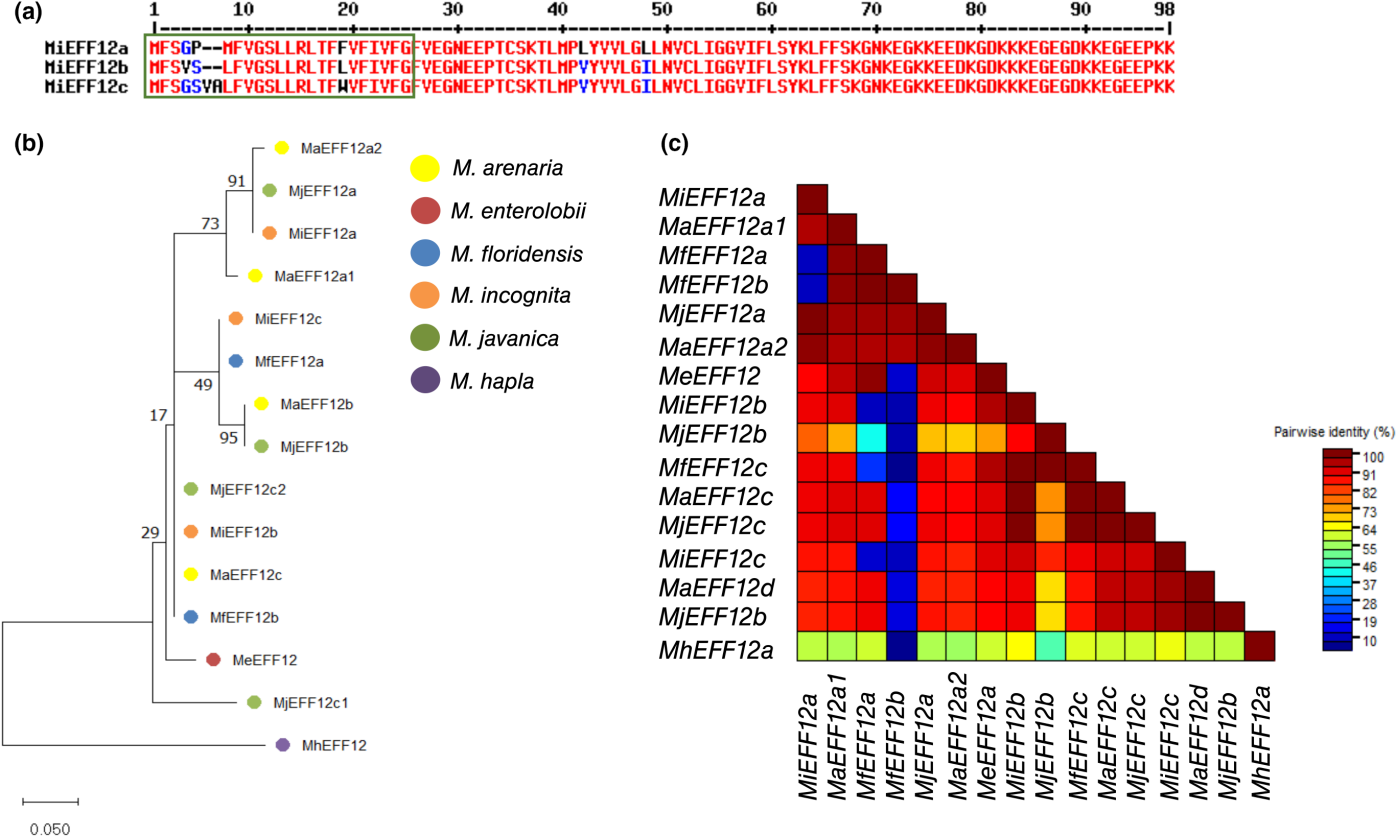
Effector 12 (EFF12) is a conserved effector in root‐knot nematodes. (a) Alignment of the MiEFF12 protein sequences. The green box indicates the position of the signal peptide for secretion. The conserved C‐terminal region is enriched in positively (lysine, K) and negatively (asparagine, D and glutamic acid, E) charged residues. (b) Phylogenetic tree of *Meloidogyne* spp. EFF12 amino acid sequences. The percentages displayed next to each branch represent the number of tree replicates in which the associated taxa were assembled in 100 bootstraps. The lengths of the branches are not proportional to phylogenetic distance. (c) Pairwise sequence identity matrix for root‐knot nematode *EFF12* nucleotide sequences.

MiEFF12s are putative secreted effectors encoded by the *M* *iEFF12a*/*Minc3s00905g18741/Minc12754* gene and its two paralogues, *MiEFF12b/Minc3s01322g22768/Minc13608* and *MiEFF12c/Minc3s00876g18368/Minc01345* (Abad et al., 2008; Blanc‐Mathieu et al., 2017) in *M. incognita*. MiEFF12a, MiEFF12b and MiEFF12c are 96‐ to 98‐amino acid (aa) proteins with a 23‐ to 25‐aa signal peptide (SP) for secretion (Figure 1a). Secreted 73‐aa MiEFF12 proteins display no similarity to any other sequence in the genus *Meloidogyne* and they carry none of the functional domains listed in public databases. The conserved C‐terminal regions are enriched in positively (lysine, K) and negatively (asparagine, D and glutamic acid, E) charged residues. A protein BLAST search with Wormbase (Howe et al., 2016) identified one EFF12 homologue in *M. hapla* and four in *M. arenaria* and *M. javanica* (Figure 1b,c). A phylogenetic tree constructed from an alignment of 15 EFF12 protein sequences and a pairwise analysis of nucleotide sequence identity revealed variations between copies within the same species and identified MhEFF12 as the most divergent of these effectors (Figure 1b,c; Figures S1 and S2). These results indicate that EFF12 is an RKN‐specific effector. *MiEFF12* genes are more strongly expressed at the juvenile parasitic stages than at the J2 preparasitic stage, suggesting that EFF12 may play a role in parasitism (da Rocha et al., 2021; Nguyen et al., 2018).

**FIGURE 2:**
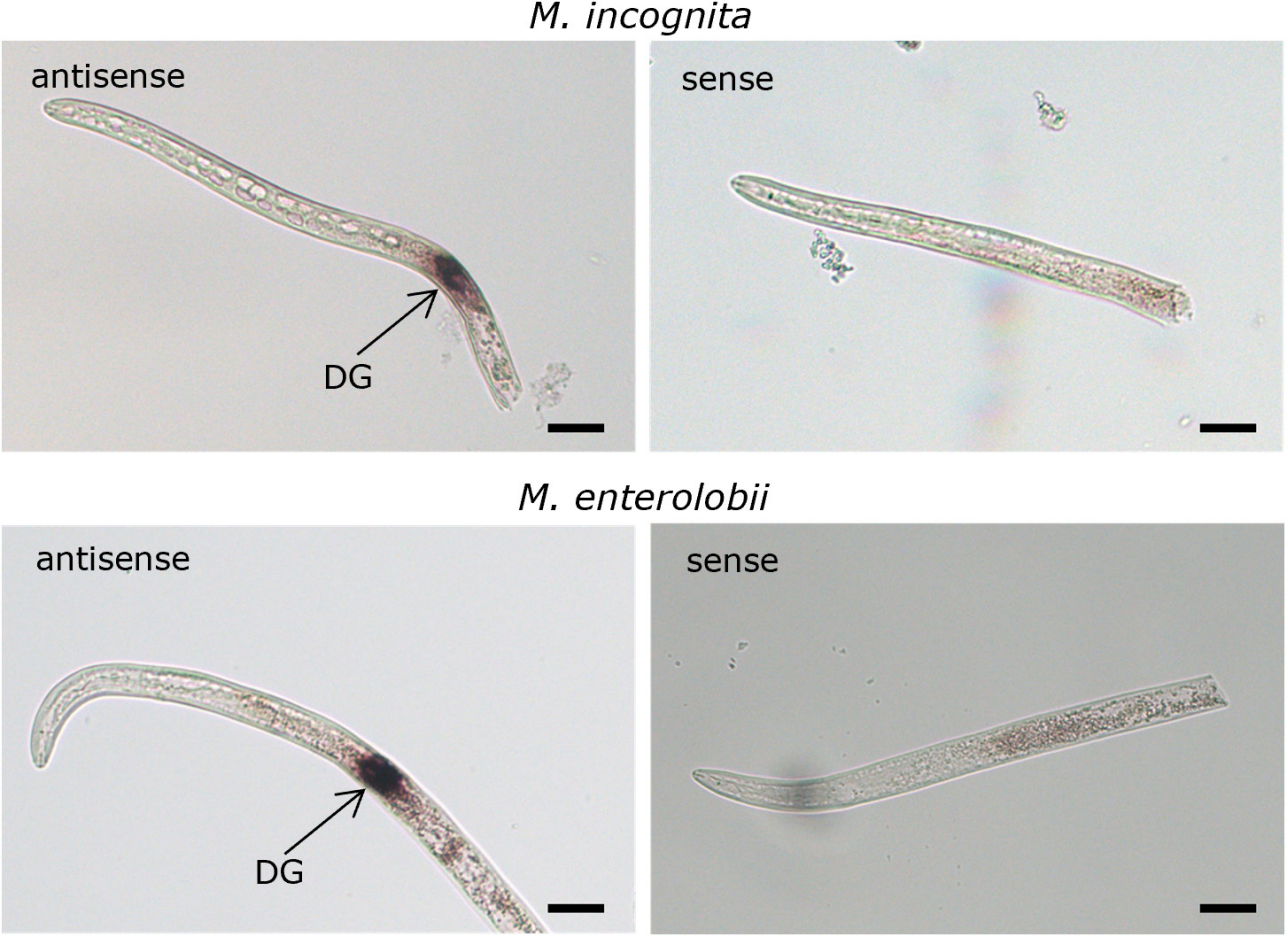
The *Meloidogyne EFF12* genes are specifically expressed in the dorsal oesophageal gland. In situ hybridization with specific antisense probes localized *EFF12* transcripts exclusively in the dorsal gland cell of preparasitic juveniles of *Meloidogyne incognita* and *Meloidogyne enterolobii*. Sense probes for *MiEFF12* and *MeEFF12* transcripts were used as a negative control and gave no signal. DG, dorsal gland. Bars: 20 μm.

We analysed the possible secretion of EFF12 into the plant during parasitism, by using in situ hybridization to localize *EFF12* expression in nematode J2s. *MiEFF12* expression was observed exclusively in the dorsal oesophageal gland of preparasitic *M. incognita* J2s, consistent with previous findings (Nguyen et al., 2018). A similar expression pattern, restricted to the dorsal gland cell, was observed in *M. enterolobii* with an antisense *MeEFF12* probe (Figure 2). Sense probes were used as negative controls and gave no staining in *M. incognita* or *M. enterolobii* (Figure 2).

**FIGURE 3:**
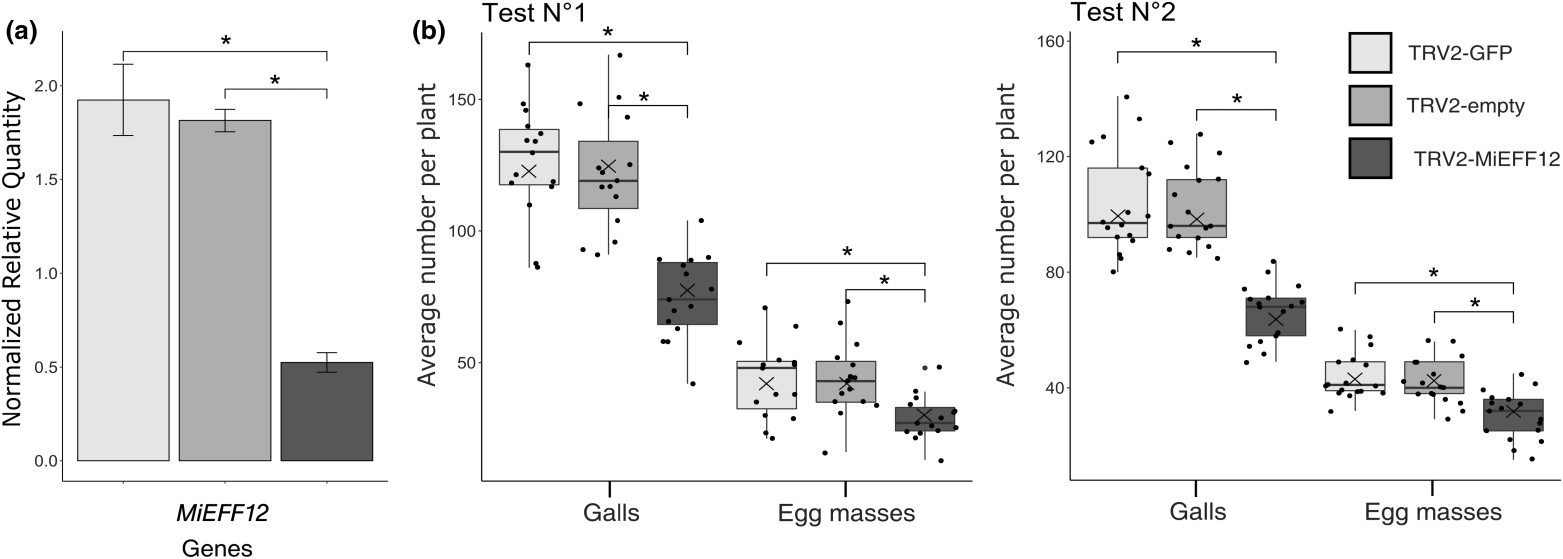
The silencing of *MiEFF12* genes by virus‐induced gene silencing affects *Meloidogyne incognita* parasitism. (a) Transcript quantification by reverse transcription‐quantitative PCR confirmed the effective silencing of *MiEFF12* genes in parasitic nematodes extracted from *Nicotiana benthamiana* roots infected with TRV2‐MiEFF12 relative to controls (TRV2‐empty and TRV2‐GFP). Normalized relative transcript levels for three independent biological replicates are shown. (b) Infection test on *N. benthamiana* control plants (TRV2‐empty and TRV2‐GFP) and plants producing siRNA for the silencing of *MiEFF12* genes in *M. incognita* (TRV2‐MiEFF12). Galls were counted 6 weeks after inoculation with 200 *M. incognita* second‐stage juveniles per plant. Results from two independent experiments are shown (*n* = 15 and *n* = 17 plants for tests 1 and 2, respectively). The cross represents average value. Box indicates interquartile range (25th to the 75th percentile). The central line within the box represents mean value. Whiskers indicate the minimum and maximum values for the normal values present in the dataset. Statistical significance was assessed in Student's *t* tests. Significant differences were observed between controls and TRV‐MiEFF12 plants (*p < 0.05).

We investigated the role of MiEFF12 in parasitism through a host‐induced gene‐silencing approach. We silenced *MiEFF12* during *M. incognita* feeding on *N. benthamiana*, using the tobacco rattle virus (TRV) for virus‐induced gene silencing (VIGS). An empty VIGS construct and a construct targeting the green fluorescent protein (*GFP*) transcript were used as controls. Reverse transcription‐quantitative PCR (RT‐qPCR) analyses showed lower levels of *MiEFF12* mRNA in nematodes collected from *N. benthamiana* roots relative to the two controls (Figure 3a). Relative to the two controls, the silencing of *MiEFF12* significantly decreased the numbers of both galls and egg masses produced by *M. incognita* on *N. benthamiana* roots 6 weeks postinfection (Figure 3b). These results demonstrate that MiEFF12 is an effector involved in parasitism.

### 2.2 MiEFF12 modulates plant immune responses

**FIGURE 4:**
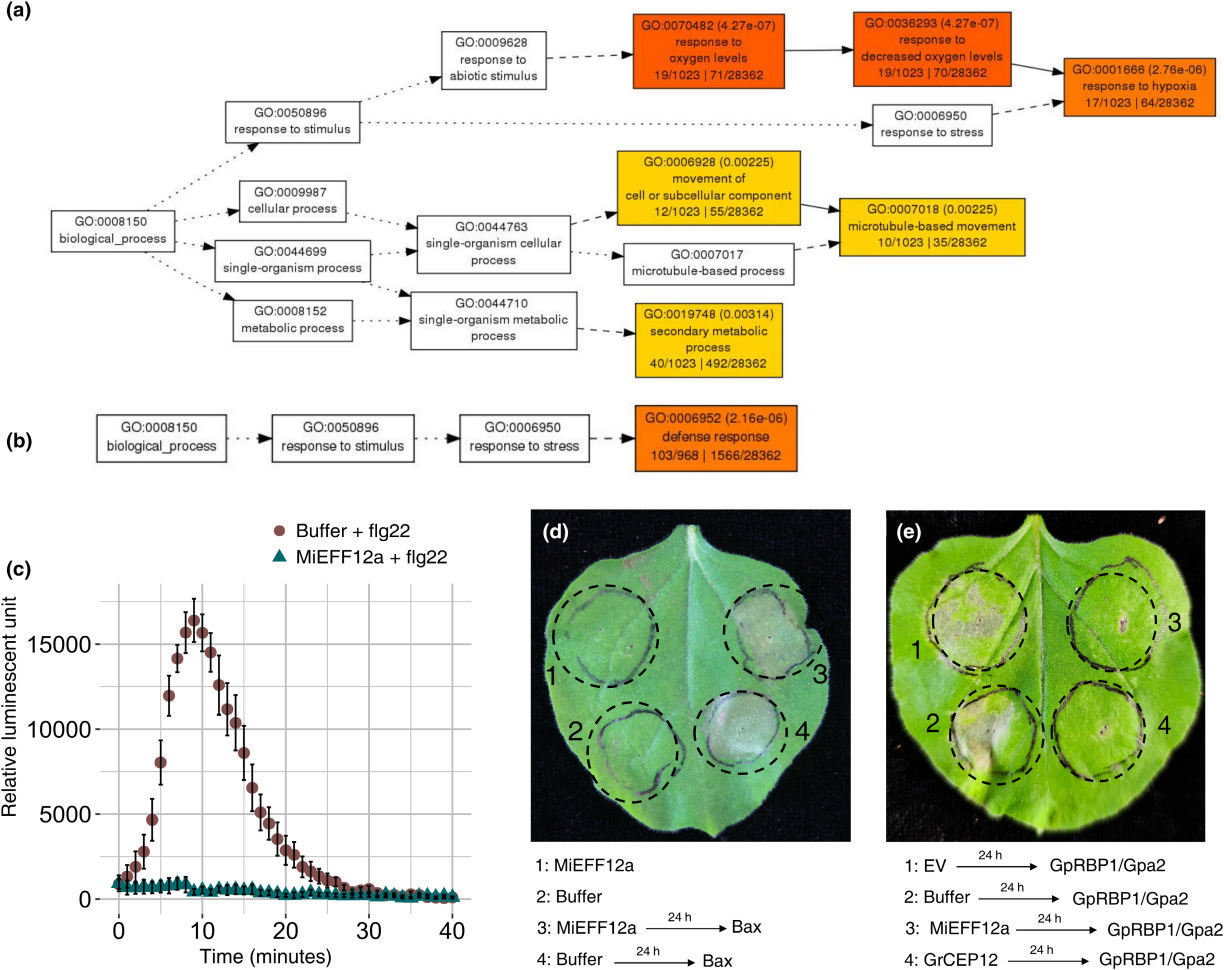
MiEFF12 suppress host defence responses. (a, b) Gene ontology (GO) enrichment analysis of differentially expressed genes (DEGs) in the *Arabidopsis MiEFF12a*‐expressing line with AgriGO v. 2.0. (a) GO enrichment analysis for the 1103 genes upregulated in the *MiEFF12a*‐expressing line with log_2_ fold change ≥1, indicating an enrichment in genes related to the response to decreased oxygen levels. (b) GO enrichment analysis on the 1126 genes downregulated in the *MiEFF12a*‐expressing line with log_2_ fold change ≤ −1, indicating an enrichment in genes related to the defence response. (c) MiEFF12 suppresses flg22‐mediated reactive oxygen species (ROS) production in *Nicotiana benthamiana*. *Agrobacterium tumefaciens* GV3101 carrying *MiEFF12a* was used to infiltrate the leaves of *N. benthamiana* plants. Infiltrated leaf discs were collected 48 h post‐agroinfiltration and assayed for ROS production in response to treatment with the flg22 elicitor. ROS production was monitored for 40 min, and the values shown are the mean relative luminescence units ± *SD* for 28 leaf discs. (d) BAX‐triggered cell death was not suppressed by MiEFF12a. Photographs for assessment of the cell‐death phenotype were taken 5 days after the last infiltration. (e) Gpa2/RBP‐1‐triggered cell death was suppressed by MiEFF12a. Photographs showing the suppression of cell death were taken 5 days after the last infiltration. Each cell death suppression bioassay was performed at least three times; results from a representative experiment are shown.

Transgenic *Arabidopsis thaliana* lines overexpressing *MiEFF12a* were generated. A large‐scale investigation of the possible effects of MiEFF12 secretion on root physiology in planta was performed by sequencing the transcripts of wild‐type (Col‐0) and *MiEFF12*‐expressing *Arabidopsis* (line #C3; Figure S3) roots. The RNA‐sequencing data analysis identified 4080 differentially expressed genes (DEGs) (adjusted *p*‐value ≤0.01). By analysing DEGs with a log_2_ fold change (FC) of expression ≥1 or ≤ −1, we found that 1103 genes were upregulated and 1126 were downregulated in the roots of the *MiEFF12*‐expressing line relative to the wild type (Table S1). A gene ontology (GO) enrichment analysis with AgriGO v. 2.0 showed that genes associated with the GO biological process “response to decreased oxygen level” were over‐represented among the DEGs upregulated in the *MiEFF12*‐expressing line (*p* = 4.3e−7; Figure 4a; Tables S2 and S3). Thirteen of the 19 genes associated with this GO term were also upregulated in galls induced by *M. incognita* in *A. thaliana* (Table S3; Yamaguchi et al., 2017). Genes involved in “defence response” were over‐represented among the 1126 DEGs downregulated in the *MiEFF12a*‐expressing line (*p* = 2.2e−6; Figure 4b; Tables S4 and S5). Most of the 103 genes associated with this GO term and downregulated in *MiEFF12*‐expressing *Arabidopsis* encoded nucleotide‐binding site (NBS) leucine‐rich repeat (LRR) proteins, LRR‐receptor kinases such as the EF‐TU receptor (EFR), receptor‐like proteins (RLPs) and PR proteins. These 103 genes included 48 known to be downregulated in galls induced by *M. incognita* (Table S5; Yamaguchi et al., 2017).

Given the role of reactive oxygen species (ROS) in cellular signalling to initiate plant immune responses, we assessed the ability of MiEFF12a to affect the ROS burst induced by the bacterial PAMP flg22 responsible for triggering PTI (Lee et al., 2020). We quantified H_2_O_2_ in a previously described luminol‐based assay (Zhao et al., 2021), in agroinfiltrated *N. benthamiana* leaves with and without *MiEFF12a* expression, after treatment with flg22 or mock treatment. Almost no H_2_O_2_ production was detected in plant leaves expressing *MiEFF12a*, whereas a ROS burst was observed in the negative control following treatment with flg22 (Figure 4c). Thus, MiEFF12a abolished the H_2_O_2_ production associated with PTI.

We also investigated whether MiEFF12a could suppress the programmed cell death physiologically resembling the HR triggered by the mouse pro‐apoptotic protein BAX (Lacomme & Santa Cruz, 1999). BAX constructs were introduced into *N. benthamiana* leaves by agroinfiltration 24 h after MiEFF12 or the control construct. As observed for the control, no inhibition of BAX‐induced apoptosis was observed when a GFP‐MiEFF12a fusion was expressed in the plant (Figure 4d; Figure S3). ETI assays were also performed with the *Globodera pallida* GpRBP‐1 protein and the potato Gpa2 resistance protein. GpRBP‐1 is recognized by Gpa2 when co‐expressed in *N. benthamiana*, triggering an HR (Sacco et al., 2009; Figure 4d). The induction of the Gpa2/GpRBP‐1‐mediated HR was suppressed by the co‐expression of MiEFF12a in *N. benthamiana* leaves (Figure 4e; Figure S3). MiEFF12a suppressed the HR as efficiently as the *Globodera rostochiensis* effector GrCEP12 used as a control (Figure 4e; Chronis et al., 2013). These findings indicate a possible role for MiEFF12 in suppressing plant immunity during plant–nematode interactions.

### 2.3 MiEFF12 targets the host cell endoplasmic reticulum

**FIGURE 5:**
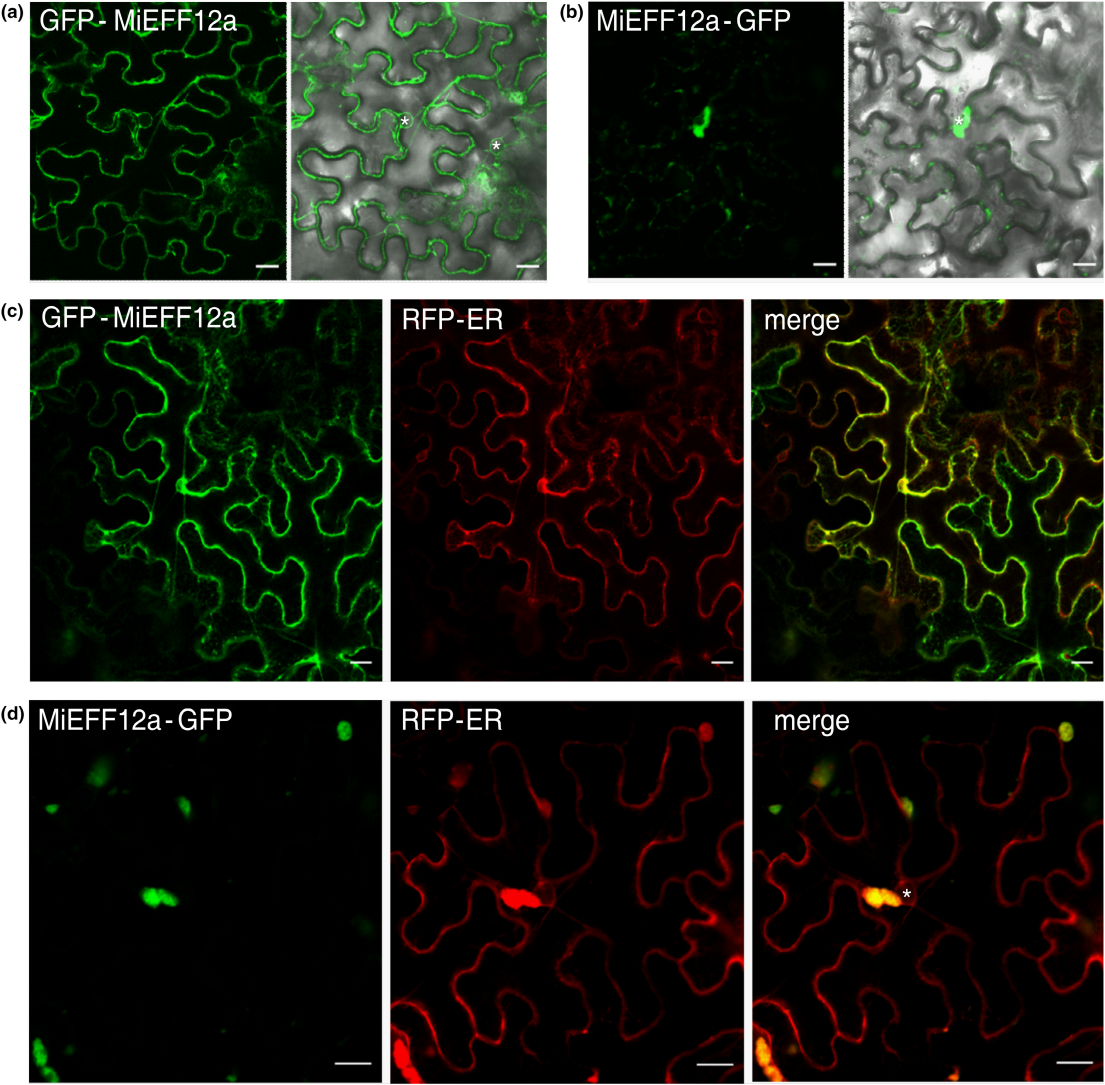
MiEFF12a was localized to the endoplasmic reticulum (ER) of epidermal *Nicotiana benthamiana* leaf cells. (a) Single‐plane confocal images of *N. benthamiana* leaf cells infiltrated with *Agrobacterium tumefaciens* and producing MiEFF12a without its signal peptide, fused to the C‐terminal end of the green fluorescent protein (GFP) reporter (GFP‐MiEFF12a; green signal; left pictures). Overlays of differential interference contrast and fluorescence images are shown (right pictures). (b) Single‐plane confocal images of *N. benthamiana* leaf cells infiltrated with *A. tumefaciens* and producing MiEFF12a without the signal peptide, fused to the N‐terminal end of the green fluorescent protein (GFP) reporter (MiEFF12a‐GFP; green signal; left pictures). Overlays of differential interference contrast and fluorescence images are shown (right pictures). (c) The monomeric red fluorescent protein (mRFP) signal (RFP‐ER; red signal) of an ER marker was used to analyse colocalization of the GFP‐MiEFF12a fusion (green signal) and the ER. (d) The mRFP signal (red signal) of the ER marker was used to analyse its colocalization with the MiEFF12a‐GFP fusion (green signal). Both fusions between the GFP and MiEFF12a colocalized with the ER marker in *N. benthamiana* leaf cells. Asterisk; nucleus. Scale bars: 20 μm.

The subcellular localization of proteins can help to elucidate their function. We localized EFF12 in plant cells by performing transient expression assays in *N. benthamiana* leaves. The coding sequence (CDS) of *MiEFF12a* without the signal peptide (SP) was transiently expressed as an N‐ or C‐terminal fusion to GFP under control of the CaMV 35S promoter. The GFP‐MiEFF12a fusion was detected in the ER—visualized as a reticulated network at the cell periphery and around the nucleus (Figure 5a). Intriguingly, the signal for the MiEFF12a‐GFP fusion was different, displaying localization within large perinuclear structures (Figure 5b). We studied the localization of MiEFF12a further, using an RFP‐ER marker (Nelson et al., 2007). With this marker, both the GFP fusions were found to colocalize with the ER marker (Figure 5c,d). The use of the RFP‐ER marker confirmed that the MiEFF12a‐GFP signal associated with perinuclear structures was indeed associated with the ER, the distribution of which was disturbed by the presence of the effector (Figure 5d). These findings indicate that MiEFF12 is an effector targeting the ER, the structure and/or function of which it is capable of altering.

### 2.4 MiEFF12a interacts with ER‐associated proteins

We investigated the function of the MiEFF12 effector in manipulating host cell physiology in more detail by performing a yeast two‐hybrid (Y2H) screen to identify direct interactors in tomato. We used MiEFF12a without its SP as a bait, and a tomato root cDNA library from healthy and *M. incognita*‐infected roots as the prey (Hybrigenics Service, France), as previously described (Mejias et al., 2021; Zhao et al., 2020). We screened 100 million interactions between MiEFF12 and the cDNA library. We identified two major effector targets, the *Solanum lycopersicum* plant bap‐like proteins (SlPBL1 and SlPBL2) and the basic region/leucine zipper motif 60 (SlBZIP60), which were captured 12 and 7 times, respectively (Table S6 and Figure S4). Other selected clones carried putative targets captured five times or less (Table S6). Both PBL and BZIP60, known ER‐associated proteins involved in ERQC, the ERAD and/or UPR systems (Atabekova et al., 2017; Liu & Howell, 2016), were considered in subsequent analyses.

**FIGURE 6:**
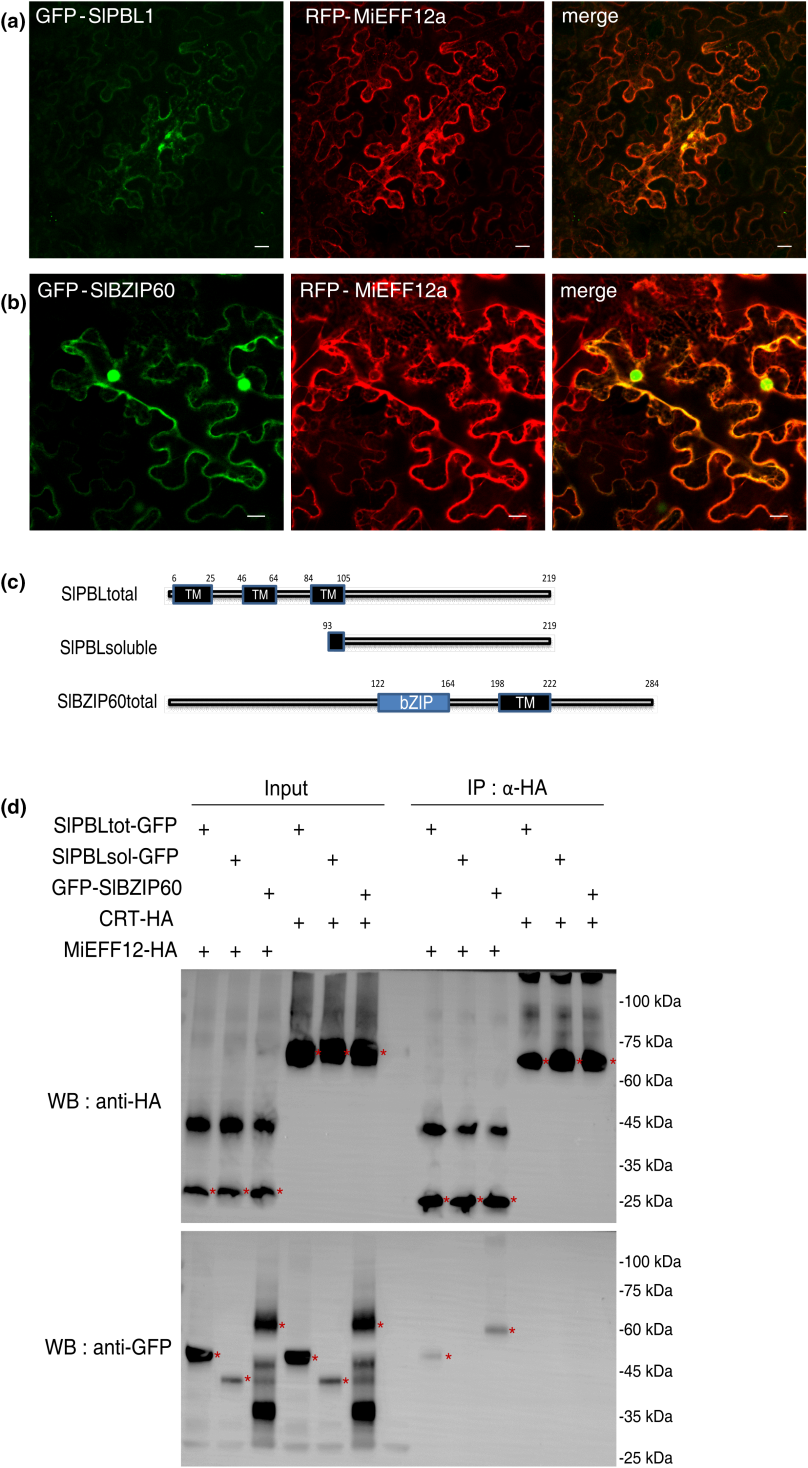
MiEFF12a physically interacts in planta with SlPBL1 and SlBZIP60. SlPBL1 and SlBZIP60 colocalized with MiEFF12a in the endoplasmic reticulum (ER) of epidermal *Nicotiana benthamiana* leaf cells. (a) Single‐plane confocal images of *N. benthamiana* leaf cells infiltrated with *Agrobacterium tumefaciens* and producing SlPBL1, fused to the C‐terminal end of the green fluorescent protein (GFP) reporter (GFP‐SlPBL1; green signal) and MiEFF12a fused to the C‐terminal end of the red fluorescent protein (RFP) reporter (RFP‐MiEFF12a; red signal). (b) Single‐plane confocal images of *N. benthamiana* leaf cells infiltrated with *A. tumefaciens* and producing SlBZIP60, fused to the C‐terminal end of the green fluorescent protein (GFP) reporter (GFP‐SlBZIP60; green signal) and the RFP‐MiEFF12a recombinant protein (RFP‐MiEFF12a: red signal). Overlays of fluorescence images are shown (merge). Scale bars: 20 μm. (c) Schematic representation of the full‐length (tot, total) and truncated (sol, soluble) SlPBL1 and SlBZIP60 proteins used for interactomic assays. (d) Co‐immunoprecipitation (Co‐IP) experiments confirmed that MiEFF12a interacted with the full‐length SlPBL1 and BZIP60. SlPBLtot‐GFP, SlPBLsol‐GFP or SlBZIP60 were transiently co‐expressed with MiEFF12a‐HA or MiCRT in *N. benthamiana* leaves. The Co‐IP experiment was performed with anti‐HA affinity gel, and the protein isolated was analysed by western blotting (WB) with anti‐GFP antibodies to detect SlPBLtot and SlPBLsol, and with anti‐HA antibodies to detect MiEFF12a and MiCRT. Three independent experiments were performed, with similar results.

Proteins present in the same subcellular compartment are more likely to be true interactors than those found in different compartments. We, therefore, performed agroinfiltration experiments to investigate the subcellular distribution of SlPBL1 and SlBZIP60 in *N. benthamiana* epidermal leaf cells. In *S. lycopersicum*, five genes—*SlPBL1* (*Solyc12g005910*), *SlPBL2* (*Solyc10g053910*), *SlPBL3* (*Solyc09g059570*), *SlPBL4* (*Solyc02g032930*) and *SlPBL5* (*Solyc02g080870*)—encode PBL proteins (Atabekova et al., 2017), whereas SlBZIP60 is encoded by a single gene, *Solyc04g082890* (Kaur & Kaitheri Kandoth, 2021) (Figure S5). The full‐length CDS of *SlPBL1* and *SlBZIP60* were cloned to generate fusion proteins with GFP. Co‐expression experiments confirmed the colocalization of RFP‐MiEFF12 with GFP‐SlPBL1 and GFP‐BZIP60 in the ER (Figure 6a,b). Unlike RFP‐MiEFF12 and GFP‐SlPBL1, GFP‐SlBZIP60 was also observed in the nucleoplasm (Figure 6b). For the SlPBL1‐GFP fusion, fluorescence was observed in large perinuclear structures similar to those observed for the MiEFF12a‐GFP fusion, while SlBZIP60‐GFP was mostly detected in the nucleoplasm (Figure S6). These results confirm that SlPBL and SlBZIP60 are located in the ER and could interact with MiEFF12 in planta.

Co‐immunoprecipitation assays (Co‐IP) were then performed to validate these interactions. The full‐length SlPBL1 and its soluble fragment and the full length of SlBZIP60 were fused separately with GFP to generate the SlPBLtotal‐GFP, SlPBLsoluble‐GFP and GFP‐SlBZIP60 constructs, respectively (Figure 6c). These constructs were co‐expressed together with MiEFF12a carrying an HA tag (MiEFF12a‐HA) in *N. benthamiana* leaves. The complete SlPBL1 and SlBZIP60 proteins were co‐immunoprecipitated with MiEFF12a (Figure 6d). Furthermore, because RKN calreticulin (CRT) effectors also localize in the ER when expressed in plant cells (Jaouannet et al., 2013; Liu et al., 2024), we used the *M. incognita* MiCRT1 effector as a negative control. MiCRT1 was unable to interact with the full‐length SlPBL1 or with its cytosolic fragment, nor with SlBZIP60 (Figure 6d). These findings demonstrate that MiEFF12a interacts with SlPBL1 and SlBZIP60 at the ER of the plant cell.

### 2.5 *PBL* s promote RKN parasitism

**FIGURE 7:**
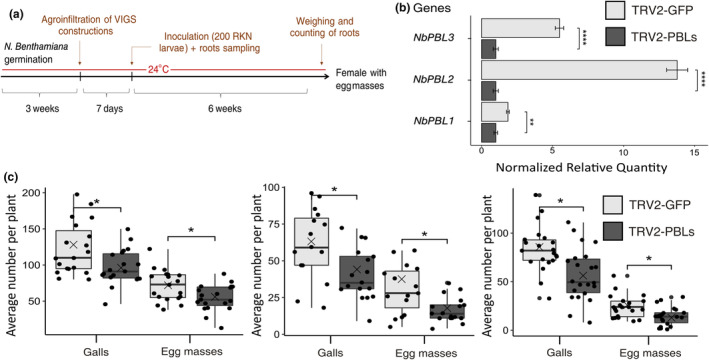
The silencing of *PBL* genes in *Nicotiana benthamiana* affects susceptibility to *Meloidogyne incognita*. (a) Timeline used for virus‐induced gene silencing (VIGS) experiments. (b) Reverse transcription‐quantitative PCR showing the efficient silencing of the *NbPBL1a/b*, *NbPBL2a/b* and *NbPBL3a/b* gene pairs in *N. benthamiana* control plants (TRV‐GFP) and plants in which *NbPBLs* were silenced (TRV2‐PBLs). The data shown are normalized relative transcript levels for three independent biological replicates obtained with SatqPCR software. The *NbEF1a* and *NbGADPH* housekeeping genes were used for data normalization. Error bars indicate the *SEM*. (c) Infection test on *N. benthamiana* TRV‐GFP or TRV2‐PBLs plants. Galls were counted 6 weeks after inoculation with 200 *M. incognita* second‐stage juveniles (J2s) per plant. Results from three independent experiments are shown (*n* = 19, *n* = 15 and *n* = 21 plants for tests 1, 2 and 3, respectively). The cross represents average value. Box indicates interquartile range (25th to the 75th percentile). The central line within the box represents mean value. Whiskers indicate the minimum and maximum values for the normal values present in the dataset. Statistical significance was determined in Student's *t* test and significant differences were observed between TRV‐GFP control and TRV‐PBL plants (*p < 0.01).

We then investigated the possible role of PBL and BZIP60 proteins in the plant immune response and RKN parasitism. A knockout *bzip60 Arabidopsis* mutant line was challenged with *M. incognita*. Six weeks after inoculation, we observed no significant effect of the *BZIP60* mutation on the number of females producing egg masses (Figure S7a). Similarly, silencing the unique *NbBZIP60* gene in *N. benthamiana* (*Niben101Scf24096g00018*) through a VIGS approach did not affect RKN development or reproduction (Figures S7b and S8). These results indicate that plant BZIP60 is not required for RKN parasitism. We then silenced *PBL* genes in *N. benthamiana* using the TRV for VIGS. Using the SGN VIGS tool (Fernandez‐Pozo et al., 2015), we selected six genes encoding NbPBLs in *N. benthamiana*: *NbPBL1a* (*Niben101Scf02543g02013*), *NbPBL1b* (*Niben101Scf08039g00007*), *NbPBL2a* (*Niben101Scf00435g05003*), *NbPBL2b* (*Niben101Scf04477g03012*), *NbPBL3a* (*Niben101Scf02516g00006*) and *NbPBL3b* (*Niben101Scf02145g09007*) (Figure S9). Using a Co‐IP approach, we verified that MiEFF12a could indeed interact with NbPBL1a, NbPBL2a and NbPBL3a (Figure S10). We then designed a chimeric TVR2 construct to silence all *NbPBL* genes. This construct specifically targeted a 200‐nucleotide region of each *NbPBL1a/b*, *NbPBL2a/b* and *NbPBL3a/b* pair (Figure S11). A TRV2 targeting a GFP transcript was used as a negative control. The TRV1 and TRV2 were introduced into 3‐week‐old *N. benthamiana* plants with *Agrobacterium tumefaciens*. Seven days after inoculation (dai), root samples were harvested for RNA extraction, and the remaining plants were inoculated with 200 *M. incognita* J2s (Figure 7a). RT‐qPCR was performed to confirm that the targeted *NbPBL* genes in the treated *N. benthamiana* plants were effectively silenced relative to the control (Figure 7b). The plants were recovered 6 weeks postinfection with *M. incognita*. The plants displayed no macroscopic developmental phenotype, and root weight was not altered by the silencing of *NbPBL* genes (Figure S12). Roots were stained with eosin, and galls and egg masses were counted. In three independent experiments, the numbers of galls and egg masses were found to be significantly smaller in plants with silenced *PBL* genes than in controls (Figure 7c). To uncover PBL involvement in regulating plant response to RKN infection, we analysed SlPBL1 function in suppressing plant defences as described earlier. SlPBL1 could suppress the ROS burst triggered by fgl22 in *N. benthamiana* (Figure S13). These results suggest that RKNs hijack the function of the plant PBL proteins, negative regulators of plant defence, to promote host susceptibility to *M. incognita*.

## 3 DISCUSSION

### 3.1 MiEFF12 is an effector targeting the host ER and involved in defence suppression

Numerous RKN effectors have been reported to suppress plant immunity (Rutter et al., 2022; Vieira & Gleason, 2019). MiCRT, a calreticulin secreted into the host apoplast, was the first RKN effector shown to suppress PTI‐triggered callose deposition and the induction of PR genes (Jaouannet et al., 2013). Several PTI‐suppressing effectors have since been reported, including *M. incognita* MiMIF‐2, MiCTL1 and MiPDCD6, and *M. graminicola* MgMO289 (Kamaruzzaman et al., 2023; Song et al., 2021; Zhao et al., 2020, 2021). RKN effectors have also been implicated in the suppression of ETI‐type cell death. The effectors involved include *M. enterolobii* MeTCTP, *M. javanica* Mj10A08 and Mimsp40, which suppress the programmed cell death triggered by the pro‐apoptotic Bcl‐2 family protein BAX (Hu et al., 2022; Niu et al., 2016; Zhuo et al., 2017). The co‐expression of a resistance gene with the corresponding avirulence effector is often used to trigger an HR in such bioassays (Naalden et al., 2018; Nguyen et al., 2018). Low levels of cell death via the HR are triggered by the recognition of *G. pallida* RBP1 proteins by the potato Gpa2 (Carpentier et al., 2013), which can be suppressed by some RKN effectors, such as *M. graminicola* MgMO289 (Song et al., 2021).

We describe here a conserved RKN effector, EFF12, that is produced in the dorsal gland. Despite the absence of a known functional domain, MiEFF12 suppresses the ROS burst induced by the flg22 peptide and the HR triggered by co‐expression of the GpRBP1 effector and the potato Gpa2 resistance protein. However, like the HR‐suppressing MiSGCR1 effector (Nguyen et al., 2018), MiEFF12 was unable to suppress the strong cell death response induced by BAX. A genome‐scaled transcriptomic analysis on a *MiEFF12a*‐overexpressing *Arabidopsis* line further confirmed the function of EFF12 in repressing defence‐associated genes.

Transient expression experiments in *N. benthamiana* epidermal leaf cells demonstrated that MiEFF12, while its not having an ER‐retention signal, targets the ER. Several RKN effectors, such as MiASP2 and 6D4 (Vieira et al., 2011), have been reported to target the host apoplast, whereas others, such as MiPDI1 and MiCTL‐1, are secreted into the cytoplasm (Zhao et al., 2020, 2021), or target the nucleus, as reported for MiEFF1 and MiEFF18 (Mejias et al., 2021; Truong et al., 2021). Only a few have been reported to target the plant endomembrane system. Several pathogens are known to target the ER to ensure the successful infestation of plants (Jing & Wang, 2020) and MiEFF12 is the third RKN effector demonstrated to target this endomembrane compartment. Kumar et al. (2023) recently described the *M. javanica* MjShKT effector, which localizes to the ER and Golgi compartments when transiently expressed in *N. benthamiana* epidermal leaf cells. However, the host cell targets of MjShKT and the mechanism of its suppression of plant cell death have not yet been identified. MgCRT1 is another RKN effector, secreted into giant cells, that localizes in the ER during parasitism (Liu et al., 2024). MgCRT1 was shown to interact with an ER‐localized rice calmodulin‐like protein OsCML31 to regulate rice immunity and promote susceptibility to *M. graminicola*.

### 3.2 PBL is a new host cell player in RKN parasitism

We identified BZIP60 and PBLs, which are known to be resident in the ER, as the host cell targets of MiEFF12, consistent with the subcellular distribution reported here for this effector. The full‐length BZIP60 is anchored in the ER membrane, where its function remains unknown. But when ER stress is elevated, the stress sensor and activator inositol‐requiring enzyme 1 (IRE1) splices BZIP60 mRNA, resulting in the production of a transcription factor that can be translocated in the nucleus allowing the UPR to restore ER homeostasis. Roles for IRE1 and BZIP60 in plant immunity have been documented (Bao & Howell, 2017; Jing & Wang, 2020). Our results indicate that suppressing BZIP60 function does not alter plant's susceptibility to RKN. The role of other UPR branches, having overlapping functions with IRE1/BZIP60 pathway, in this biotrophic interaction remains, however, to be investigated. B‐cell receptor‐associated protein 31 (BAP31) is a conserved integral ER‐associated protein with three transmembrane domains in its N‐terminal region and a C‐terminal cytosolic tail (Quistgaard, 2021). In animals, BAP31 has been described as an important chaperone or quality control factor involved in ER membrane protein sorting, promoting protein retention in the ER, transport from the ER to the downstream secretory pathway or other cellular compartments, or targeting to the ERAD system for degradation (Quistgaard, 2021; Wakana et al., 2008). It is also cleaved by caspase and plays a role in apoptosis (Breckenridge et al., 2003; Quistgaard, 2021). The equivalent proteins to BAP31 in plants are probably BAP‐like proteins (PBLs) and are encoded by multigene families (Atabekova et al., 2017). The functions of PBLs remain largely undescribed. A PBL from *Nicotiana tabacum*, NtPBL, was shown to interact with the tobacco protein Nt‐4/1, a protein known to interact with plant virus movement proteins and to affect the long‐distance transport of potato spindle tuber viroid (PSTVd) via the phloem (Pankratenko et al., 2017). Interestingly, NtPBL was shown to induce the relocalization of Nt‐4/1 to ER bodies and large aggregates with a granular structure (Pankratenko et al., 2017) similar to those we observed when overexpressing MiEFF12a‐ or SlPBL1‐GFP fusions. In animal cells, the relocalization of BAP31 under stress conditions to a juxtanuclear ER compartment involved in the ER‐associated degradation of misfolded proteins is well documented (Quistgaard, 2021). In addition, the cytosolic tail of NtPBL has been shown to bind RNA (pre‐miRNA and viroid RNA), and the expression of *NtPBL* in *N. benthamiana* via a TRV‐based approach strongly affects plant development and/or the symptoms induced by TRV (Atabekova et al., 2017). We show here that NbPBLs play a role in RKN parasitism, as plants in which *NbPBL* genes were silenced were less susceptible to *M. incognita*. Furthermore, our finding indicates that PBLs negatively regulate host immunity.

### 3.3 MiEFF12 targets plant PBLs to suppress ER‐mediated immunity

The ER plays an important role in plant immunity. PRRs are synthesized in the ER and are subject to ER quality control to ensure that they are transported in the correctly folded form from the ER to the plasma membrane (Park & Seo, 2015). As an example, actors of the ER quality control were shown to be involved in the EF‐Tu receptor (EFR) biogenesis (Li et al., 2009; Nekrasov et al., 2009). Similarly, antimicrobial PR proteins secreted following recognition of the infecting pathogens are produced and folded at the ER and then processed by the Golgi apparatus for delivery to the apoplast. As a result, the ER is frequently targeted by pathogens and several ER‐associated proteins have been implicated in plant responses to pathogen infections (Jing & Wang, 2020). Given the role of BAP31 as a chaperone controlling the fate of the protein—retention in the ER, export, degradation by the ERAD or escape from degradation (Quistgaard, 2021)—we suggest that these functions are conserved in the PBLs of plants and are hijacked by MiEFF12 to prevent the initiation or full expression of PTI. We have shown that MiEFF12a can suppress HR‐cell death, possibly through its interaction with SlPBLs. In plants, the ER, or more specifically ER stress, is a recognized initiator of programmed cell death (Eichmann & Schäfer, 2012; Simoni et al., 2022). The role of PBLs in modulating these pathways remains to be elucidated, but the induction of plant cell death programmes must be prevented if biotrophic interactions are to be initiated and maintained throughout the nematode life cycle. Several molecules have already been identified as involved in both PTI and ETI, and PBLs may play such a role in the interaction of the PTI and ETI signalling pathways.

Further analysis would be required to define more precisely the functions of PBLs in regulating plant immunity and the ways in which the effector affects them. This study has taken the first step in this process by revealing a new pathogenic strategy used by RKNs to hijack plant metabolism. In this strategy, the MiEFF12 effector targets PBLs, corrupting their functions so as to promote RKN parasitism. PBL‐encoding genes, thus, emerge as promising susceptibility genes that could be targeted in innovative breeding strategies with the aim of generating RKN‐resistant crops.

## 4 EXPERIMENTAL PROCEDURES

### 4.1 Nematode and plant materials

*M. incognita* strain ‘Morelos’ was multiplied in tomato (*S. lycopersicum* ‘Saint Pierre’) grown in a growth chamber (25°C, with a 16‐h photoperiod). Freshly hatched J2s were collected as described by Caillaud and Favery (2016). The previously described *bzip60* mutant (SALK_050203; Lu & Christopher, 2008) was obtained from the Arabidopsis Biological Resource Center (ABRC, Ohio State University, Columbus, OH, USA). For VIGS experiments, *N. benthamiana* seeds were sown on soil and incubated at 4°C for 2 days. After germination, plantlets were transplanted into pots containing soil and sand (1:1) and were grown at 24°C (photoperiod, 16‐h light: 8‐h dark). For transcriptome analysis, *Arabidopsis* seeds were surface‐sterilized and added to liquid Murashige and Skoog (MS) medium (0.5 × MS salts, 1% sucrose, pH 6.4). They were incubated at 25°C, under a 12‐h period, with gentle shaking (70 rpm), as previously described (Mejias et al., 2021).

### 4.2 EFF12 sequence analysis, alignment and phylogenetic tree

The sequences of putative *EFF12* paralogues and orthologues were obtained from *Meloidogyne* genomic resources (http://www6.inra.fr/meloidogyne_incognita and Wormbase parasite). We used 15 *Meloidogyne* *EFF12* sequences from *M. arenaria*, *M. enterolobii*, *M. incognita*, *M. floridensis* and *M. javanica* in this analysis. The sequences of the proteins encoded by these genes were analysed with PHOBIUS and Prosite to identify the SP and to search for putative functional domains, respectively (http://phobius.sbc.su.se/; https://prosite.expasy.org/). EFF12 sequences were aligned with the ClustalW algorithm (Thompson et al., 2003) and their evolutionary history was inferred by maximum‐likelihood methods, as previously described (Berger et al., 2020). *M. hapla* was used as the outgroup for the phylogenetic tree based on putative orthologues of EFF12.

### 4.3 In situ hybridization

In situ hybridization was performed on freshly hatched *M. incognita* and *M. enterolobii* J2s as previously described (Jaouannet et al., 2018). For probe production, the *MiEFF12a*, and *MeEFF12* sequences were amplified specifically from entry vectors with the primers MiEFF12_GW3 and MiEFF12_GW5 (for MiEFF12a) or MeEFF12_GW3 and MeEFF12_GW5 (for MeEFF12) (Table S7). Sense probes for *MiEFF12a* and *MeEFF12* were used as negative controls. Photomicrographs were obtained with an Axioplan2 microscope (Zeiss).

### 4.4 RKN infection assay, juveniles in the plant

Three‐week‐old *Arabidopsis* seedlings were inoculated with 200 *M. incognita* J2s per plant. Roots were collected 6 weeks after infection and stained with 0.5% eosin. The number of females forming egg masses was then determined. *N. benthamiana* plants subjected to VIGS were inoculated with 200 *M. incognita* J2s per plant, 7 days postinoculation with TRV, and incubated at 24°C. Infected *N. benthamiana* roots were collected 6 weeks after infection. Galls or egg masses were counted under a binocular microscope, and the root system was weighed.

### 4.5 Subcellular distribution in the plant

The *M. incognita* MiEFF12 CDS lacking the SP, the *S. lycopersicum* PBL (total and soluble portion) and BZIP60 (unspliced form) were amplified by PCR with specific primers (Table S7) and inserted into the pDON207 donor vector. They were recombined in pK2GW7 (P35S:MiEFF12), pK7WGR2 (P35S:mRFP‐MiEFF12a), pK7FGW2 (P35S:eGFP‐SlPBL, P35S:eGFP‐SlBZIP60 and P35S:eGFP‐MiEFF12a) or pK7FWG2 (P35S:SlPBL‐eGFP, P35S:SlBZIP60‐eGFP and P35S: MiEFF12a‐eGFP) (Karimi et al., 2002) with Gateway technology (Invitrogen). All the constructs were sequenced (GATC Biotech) and transferred into *A. tumefaciens* GV3101. Leaves from 3‐ to 4‐week‐old *N. benthamiana* plants were subjected to agroinfiltration with recombinant strains of *A. tumefaciens* containing GFP or RFP vectors as described by Mejias et al. (2021). Leaves were imaged 48 h after agroinfiltration, with an inverted confocal microscope (LSM 880; Zeiss) equipped with an argon ion laser as the excitation source. Samples were excited at 488 nm for GFP and 543 nm for RFP. GFP and RFP emissions were detected selectively with 505–530 and 560–615 nm band‐pass emission filters respectively.

### 4.6 Yeast two‐hybrid screen

For the Y2H screen, the CDS of the *MiEFF12* without the SP was used as a bait. The *MiEFF12a* sequence was amplified (Table S2) and inserted into the pB27 vector (Hybrigenics Services, Paris, France). The Y2H screen was performed with an infested tomato root cDNA library (Hybrigenics Services, Paris, France) as previously described (Mejias et al., 2021).

### 4.7 Host‐induced silencing of *MiEFF12a* and virus‐induced silencing of *PBL* and *BZIP60*

Host‐induced gene silencing (HIGS) and VIGS assays were performed on *N. benthamiana*. For HIGS experiments, fragments of *MiEFF12a* (291 bp) and *GFP* (298 bp) were amplified by PCR with the primers listed in Table S7 and inserted into the TRV2 vector. HIGS experiments were performed as described by Zhao et al. (2021), and RT‐qPCR was performed to validate the silencing of the *MiEFF12* genes 10 days after inoculation with the TRV. We used the VIGS‐Tool (https://vigs.solgenomics.net/) to design the best sequence for silencing *BZIP60* and *PBL* genes in *N. benthamiana* (Figures S7 and S9). A 300‐nucleotide fragment of *NbBZIP60* was PCR‐amplified using specific primers (Table S7) before ligation into the TRV2 plasmid. The nucleotide sequences for the following pairs of genes—*NbPBL1a*/*NbPBL1b*, *NbPBL2a*/*NbPBL2b*, *NbPBL3a*/*NbPBL3b*—were sufficiently similar for the design of a chimeric construct targeting 200 nucleotides for each pair synthesized with pUC57 (Gene Universal Inc., Newark, DE, USA). The insert was then inserted into the TRV2 plasmid (Figure S9) and the resulting construct was used to transform *A. tumefaciens* GV3101. VIGS experiments were performed as previously described (Mejias et al., 2022). Each treatment involved at least 20 *N. benthamiana* plants, and the entire experiment was performed at least twice.

### 4.8 Cell death and PTI suppression assay in *N. benthamiana*

The CDS of MiEFF12 and GFP were amplified and inserted into the super1300 vector. The resulting constructs were then used to transform *A. tumefaciens* GV3101. *Agrobacterium* cells carrying BAX or GpRBP1/Gpa2 were used to trigger cell death in *N. benthamiana* leaves (Jing et al., 2016; Sacco et al., 2009). GrCEP12 was used as a positive control to suppress GpRBP1/Gpa2‐induced cell death (Chronis et al., 2013). Agroinfiltrations into 4‐week‐old *N. benthamiana* leaves were performed as described elsewhere (Nguyen et al., 2018). Photographs were taken 5 days postinoculation for the assessment of cell death.

For the ROS assays, the CDS of MiEFF12 without its native SP and SlPBL1 were inserted into the super1300 vector. Four‐week‐old *N. benthamiana* leaves were infiltrated with *A. tumefaciens* carrying the plasmid, and buffer was used for control. Two days after infiltration, leaf discs were collected and prepared for the ROS assay (luminol‐based method) as previously described (Chen et al., 2008).

### 4.9 Reverse transcription‐quantitative PCR

Total RNA was extracted with the RNeasy Mini kit (Qiagen), and cDNA was synthesized with the SuperScript III First‐Strand Synthesis system (Invitrogen) according to the manufacturer's instructions. At least three separate biological replicates were performed for each experiment. qPCR was performed with Maxima SYBR Green qPCR Master Mix (29; Fermentas, Thermo Fisher Scientific) on an I‐Cycler (Bio‐Rad) with gene‐specific primers (Table S1). Quantifications and statistical analyses were performed with SATqPCR (Rancurel et al., 2019).

### 4.10 Co‐immunoprecipitation assays

For the Co‐IP assay, the CDS of MiCRT and MiEFF12a (without the SP sequence) or SlPBL1total, SlPBL1soluble, NbPBL1a, NbPBL2a and NbPBL3a were inserted into the super1300 vectors with a HA‐tag and a GFP‐tag, respectively, fused to the C‐terminal end of the sequence, and SlBZIP60 was cloned into pBIN vector with a GFP‐tag fused to the N terminus of the sequence. Total protein was extracted from 4‐week‐old *N. benthamiana* leaves co‐expressing SlPBL1 or SlBZIP60 and MiEFF12a or MiCRT, after 48 h of infiltration. Co‐IPs were performed with BeyoMag anti‐HA Magnetic Beads (Beyotime), and the eluted proteins were identified by western blotting with anti‐GFP (Beyotime) and anti‐HA (Beyotime) antibodies as described by Zhao et al. (2023).

### 4.11 Transcriptome analysis

For RNA‐seq experiments, seeds were surface‐sterilized and sown in liquid MS medium (½ × MS salts, 1% sucrose, pH 6.4) with gentle shaking (70 rpm), under a 12‐h photoperiod, at 25°C. Roots were collected after 11 days and immediately frozen in liquid nitrogen. Total RNA was extracted with TRIzol (Invitrogen) according to the Invitrogen protocol. The RNA was treated with DNase (Ambion), and its quality and integrity were assessed with a bioanalyser (Agilent). Library construction, paired‐end sequencing and data analysis were performed as described by Mejias et al. (2021). Gene ontology enrichment analysis was performed with the agriGO v. 2.0 toolkit (http://systemsbiology.cau.edu.cn/agriGOv2/; Tian et al., 2017) using default parameters.

### 4.12 Statistical analysis

Graphs and plots were created with R and Microsoft Office Excel 2019. Statistical analyses were performed with R software (R Development Core Team, v. 4.1.0). and SATqPCR (https://satqpcr.sophia.inrae.fr/cgi/home.cgi; Rancurel et al., 2019).

## CONFLICT OF INTEREST STATEMENT

The authors have no conflict of interest to declare.

## Supporting information


**Figure S1.** Amino acid sequences of EFF12 effector proteins identified in root‐knot nematode species.

**Figure S2.** Nucleotide sequences of *EFF12‐*encoding genes identified in root‐knot nematode species.

**Figure S3.** Ectopic expression of MiEFF12a in *Arabidopsis thaliana* does not affect root development.

**Figure S4.** MiEFF12a interacts with SlPBL1, SlPBL2 and SlBZIP60 in yeast.

**Figure S5.** Nucleotide sequences of SlPBL1 and SlBZIP60.

**Figure S6.** MiEFF12a and SlPBL1 colocalize in *Nicotiana benthamiana* epidermal leaf cells and both MiEFF12a‐ and SlPBL1‐GFP fusions were localized in large subcellular structures mostly juxtanuclear.

**Figure S7.** Nucleotide sequences of *Nicotiana benthamiana* BZIP60 and design of the virus‐induced gene silencing (VIGS) construct.

**Figure S8.** Plant BZIP60 is not required for *Meloidogyne incognita* parasitism.

**Figure S9.** Nucleotide sequence of *Nicotiana benthamiana PBL* genes targeted by the virus‐induced gene silencing (VIGS) approach.

**Figure S10.** MiEFF12a physically interacts in planta with NbPBL1a, NbPBL2a and NbPBL3a.

**Figure S11.** A chimeric sequence was introduced into the TRV2 RNA to silence the NbPBL genes through virus‐induced gene silencing (VIGS).

**Figure S12.** The silencing of *PBL* genes by virus‐induced gene silencing (VIGS) does not affect *Nicotiana benthamiana* root development.

**Figure S13.** MiEFF12a and SlPBL1a suppress flg22‐mediated reactive oxygen species (ROS) production in *Nicotiana benthamiana*.

**Table S1.** Differentially expressed genes identified in the MiEFF12‐expressing *Arabidopsis* line.

**Table S2.** Gene ontology (GO) analyses of the 1103 genes upregulated in the *MiEFF12a*‐expressing line with a log_2_ fold change ≥1.

**Table S3.** List of 19 genes upregulated in the *EFF12*‐expressing line and associated with GO term “GO:0036293 response to decreased oxygen levels”.

**Table S4.** Gene ontology (GO) analyses of the 1126 genes downregulated in the *MiEFF12a*‐expressing line with log_2_ fold change ≤ −1.

**Table S5.** List of 103 genes downregulated in the *EFF12*‐expressing line and associated with GO term “GO:0006952 defense response”.

**Table S6.** Results of the yeast two‐hybrid screen using MiEFF12a as a bait against the tomato root cDNA library.

**Table S7.** Primers used in this study.

## Data Availability

Sequence data from this article can be found in Solgenomics (https://solgenomics.net/) and GenBank/EMBL databases under the following accession numbers: *Meloidogyne incognita* MiEFF12a/Minc12754 (KX907763), MiCRT/Minc06693 (AF402771.1); *Arabidopsis thaliana* AtBZIP60 (AT1G42990); *Nicotiana benthamiana* NbBZIP60 (Niben101Scf24096g00018), NbPBL1a (Niben101Scf02543g02013), NbPBL1b (Niben101Scf08039g00007), NbPBL2a (Niben101Scf00435g05003), NbPBL2b (Niben101Scf04477g03012), NbPBL3a (Niben101Scf02516g00006), NbPBL3b (Niben101Scf02145g09007); *Solanum lycopersicum* SlBZIP60 (Solyc04g082890), SlPBL1 (Solyc12g005910), SlPBL2 (Solyc10g053910), SlPBL3 (Solyc09g059570), SlPBL4 (Solyc02g032930), SlPBL5 (Solyc02g080870). The transcriptome data are available from the Sequence Read Archive (SRA) under accession numbers PRJNA641665 and PRJNA719908 (*A. thaliana* Col‐0 and P35S:MiEFF12 roots respectively). Other data supporting the findings of this study are available from the corresponding author upon reasonable request.
