# Supplementary material for: The root‐knot nematode effector MiEFF12 targets the host ER quality control system to suppress immune responses and allow parasitism
Source: Mol Plant Pathol. 2024 Jul 4;25(7):e13491. doi: 10.1111/mpp.13491 (PMC11222708; doi:10.1111/mpp.13491)
Supplement: Supplementary file 1 — Figure S1. Amino acid sequences of EFF12 effector proteins identified in root‐knot nematode species. [file MPP-25-e13491-s010.pdf]

>MiEFF12a
MFSGPMFVGSLLRLTFFVFIVFGFVEGNEEPTCSKTLMPLYVVLGLLNVCLIGGVIFLSYKLFFSKGNKEGKKEEDKGDKKKEGEGDKKEGEEPKK
>MiEFF12b
MFSVSLFVGSLLRLTFLVFIVFGFVEGNEEPTCSKTLMPVYVVLGILNVCLIGGVIFLSYKLFFSKGNKEGKKEEDKGDKKKEGEGDKKEGEEPKK
>MiEFF12c
MFSGSVALFVGSLLRLTFWVFIVFGFVEGNEEPTCSKTLMPVYVVLGILNVCLIGGVIFLSYKLFFSKGNKEGKKEEDKGDKKKEGEGDKKEGEEP
KK
>MaEFF12a1
MFSGPMFVGNLLRLTFLVFIVFGFVEGNEEPTCSKTLMPVYVVLGLLNVCLIGGVIFLSYKLFFSKGNKEGKKEEDKGDKKKEGEGDKKEGEEPK
K
>MaEFF12a2
MFSGPMFVGSLLRLTFFVFIVFGFVEGNEEPTCSKTLMSLYVVLGLLNVCLIGGVIFLSYKLFFSKGNKEGKKEEDKGDKKKEGEGDKKEGEEPKK
>MaEFF12b
MFSGSVALFVGSLLRLTFWVFIVFGFVEGKEEPTCSKTLMPVYVVLGFLNVCLIGGVIFLSYKLFFSKGNKEGKKEEDKKKEGEGDKKEGEEPKK
>MaEFF12c
MFSVSLFVGSLLRLTFLVFIVFGFVEGNEEPTCSKTLMPVYVVLGILNVCLIGGVIFLSYKLFFSKGNKEGKKEEDKKKEGEGDKKEGEEPKK
>MjEFF12a
MFSGPMFVGSLLRLTFFVFIVFGFVEGNEEPTCSKTLMPLYVVLGLLNVCLIGGVIFLSYKLFFSKGNKEGKKEEDKGDKKKEGEGDKKEGEEPKK
>MjEFF12b
MFSGSVALFVGSLLRLTFWVFIVFGFVEGKEEPTCSKTLMPVYVVLGFLNVCLIGGVIFLSYKLFFSKGNKEGKKEEDKKKEGEGDKKEGEEPKK
>MjEFF12c1
MCSVSLFVGSLLRLTFLVFIVFGFVEGNEEPTCSKTLMPVYVVLGILNVCLIGGTCSKTLMPLYVVLGLLNVCLIGGVIFLSYKLFFSKGNKEGKKEE
DKGDKKKEGEGDKKEGKNLKSKGIKCVEDIGIKCRVNFVLFFFV
>MjEFF12c2
MFSVSLFVGSLLRLTFLVFIVFGFVEGNEEPTCSKTLMPVYVVLGILNVCLIGGVIFLSYKLFFSKGNKEGKKEEDKKKEGEGDKKEGEEPKK
>MfEFF12a
MPVYVVLGILNVCLIGGVIFLSYKLFFSKGNKEGKKEEDKGDKKKEGEGDKKEGEEPKKEDVIGIKCRCCLFAKYNQKNQRLFILCIRGAGQGYPG
HRRTITSLKIVESVKP
>MfEFF12b
MPVYVVLGILNVCLIGGVIFLSYKLFFSKGNKEGKKEEDKGDKKKEGEGDKKEGEEPKK
>MeEFF12
MPSSSVSLFVGSLLRLTFLVFIVFGFVEGNEEPTCSKTLMPVYVVLGLLNVCLIGGVIFLSYKLFFSKGNKEGKKEEDKGDKKKEGEGDKKEGEEPK
K
>MhEFF12
MCSDSVSLSIGYLFRLMCLMFFVFGFVKATEPTCSQTLLPLYIGLGFLNVCLIGGIIFLSFKIFSSKGNKKEENKKEDKKENEEPKKVKE

Figure S1. Amino acid sequences of EFF12 effector proteins identified in RKN species.
