# Supplementary material for: The root‐knot nematode effector MiEFF12 targets the host ER quality control system to suppress immune responses and allow parasitism
Source: Mol Plant Pathol. 2024 Jul 4;25(7):e13491. doi: 10.1111/mpp.13491 (PMC11222708; doi:10.1111/mpp.13491)
Supplement: Supplementary file 2 — Figure S2. Nucleotide sequences of EFF12‐encoding genes identified in root‐knot nematode species. [file MPP-25-e13491-s012.pdf]

>MiEFF12a
ATGTTTTCTGGTCCTATGTTTGTTGGGAGTTTGCTTCGTTTGACATTTTTTGTGTTTATTGTTTTTGGATTTGTGGAGGGGAATGAGGAACC
GACTTGTTCAAAAACGCTTATGCCGTTATATGTTGTTTGGGACTTTTGAATGTTTGCCTTATTGGTGGCGTTATCTTTCTCTCTTACAAATTA
TTCTTTTCTAAAGGAAATAAAGAAGGAAAGAAGGAAGAAGATAAAGGAGATAAAAAGAAAGAAGGGGAAGAGATAAGAAAGAGGGG
GAAGAGCCTAAAAAGTAA
>MiEFF12b
ATGTTTTCTGTTTCTCTGTTTGTTGGGAGTTTGCTTCGTTTGACATTTTTGGTGTTTATTGTTTTTGGATTTGTGGAGGGGAATGAGGAACC
GACTTGTTCTAAAACGCTTATGCCGGTATATGTTGTTTGGGAATTTTGAATGTTTGCCTTATTGGTGGTGTTATCTTTCTCTCTTATAAATTA
TTCTTTTCTAAAGGAAATAAAGAAGGGAAGAAGGAAGAAGATAAAGGAGATAAAAAGAAGGAAGGGGAAGAGATAAGAAAGAAGGG
GAAGAACCTAAAAAGTAA
>MiEFF12c
ATGTTTTCTGGTTCTGTTGCTCTGTTTGTTGGGAGTTTGCTTCGTTTGACATTTTGGGTGTTTATTGTTTTTGGATTTGTGGAGGGGAATGA
GGAACCGACTTGTTCAAAAACGCTTATGCCGGTATTGTTGTTTTGGGAATTTTGAATGTTTGCCTTATTGGTGGTGTTATCTTTCTCTCTTA
CAAATTATTCTTTTCTAAAGGAAATAAAGAAGGGAAGAAGGAAGAAGATAAGGGAGATAAAAAGAAGGAAGGGAGGGAGATAAGAAA
GAAGGGGAAGAACCTAAAAAGTAA
>MaEFF12a1
ATGTTTTCTGGTCCTATGTTTGTTGGGAATTTGCTTCGTTTAACATTTTTGGTGTTTATTGTTTTTGGATTTGTGGAGGGGAATGAGGAACC
GACTTGTTCAAAAACGCTTATGCCGGTATATGTTGTTTGGGACTTTTGAATGTTTGCCTTATTGGTGGCGTTATCTTTCTCTCTTACAAATT
ATTCTTTTCTAAAGGAAATAAAGAAGGGAAGAAGGAAGAAGATAAAGGAGATAAAAAGAAAGAAGGGGAGGAGATAAGAAAGAAGG
GGAAGAACCTAAAAAGTAA
>MaEFF12a2
ATGTTTTCTGGTCCTATGTTTGTTGGGAGTTTGCTTCGTTTGACATTTTTTGTGTTTATTGTTTTTGGATTTGTGGAGGGGAATGAGGAACC
GACTTGTTCAAAAACGCTTATGTCGTTATATGTTGTTTGGGACTTTTGAATGTTTGCCTTATTGGTGGCGTTATCTTTCTCTCTTACAAATTA
TTCTTTTCTAAAGGAAATAAAGAAGGAAAGAAGGAAGAAGATAAAGGAGATAAAAAGAAGGAAGGGGAAGAGATAAGAAAGAGGGG
GAAGAACCTAAAAAGTAA
>MaEFF12b
ATGTTTTCTGGTTCTGTTGCTCTGTTTGTTGGGAGTTTGCTTCGTTTGACATTTTGGGTGTTTATTGTTTTTGGATTTGTGGAGGGGAAGGA
GGAACCGACTTGTTCTAAAACGCTTATGCCGGTATTGTTGTTTTGGGATTTTTGAATGTTTGCCTTATTGGTGGCGTTATCTTTCTCTCTTA
CAAATTATTCTTTTCTAAAGGAAATAAAGAAGGAAAGAAGGAAGAAGATAAAAAGAAGGAAGGGGAAGGAATAAGAAAGAAGGGGA
AGAACCTAAAAAGTAA
>MaEFF12c
ATGTTTTCTGTTTCTCTGTTTGTTGGGAGTTTGCTTCGTTTGACATTTTTGGTGTTTATTGTTTTTGGATTTGTGGAGGGGAATGAGGAACC
GACTTGTTCAAAAACGCTTATGCCGGTATATGTTGTTTGGGAATTTTGAATGTTTGTCTTATTGGTGGCGTTATCTTTCTCTCTTATAAATT
ATTCTTTTCTAAAGGAAATAAAGAAGGGAAGAAGGAAGAAGATAAAAAGAAGGAAGGGGAAGGAGATAAGAAGAAGGGGAAGAACC
TAAAAAGTAA
>MjEFF12a
ATGTTTTCTGGTCCTATGTTTGTTGGGAGTTTGCTTCGTTTGACATTTTTTGTGTTTATTGTTTTTGGATTTGTGGAGGGGAATGAGGAACC
GACTTGTTCAAAAACGCTTATGCCGTTATATGTTGTTTGGGACTTTTGAATGTTTGCCTTATTGGTGGCGTTATCTTTCTCTCTTACAAATTA
TTCTTTTCTAAAGGAAATAAAGAAGGAAAGAAGGAAGAAGATAAAGGAGATAAAAAGAAGGAAGGGGAAGAGATAAGAAAGAGGGG
GAAGAACCTAAAAAGTAA
>MjEFF12b
ATGTTTTCTGGTTCTGTTGCTCTGTTTGTTGGGAGTTTGCTTCGTTTGACATTTTGGGTGTTTATTGTTTTTGGATTTGTGGAGGGGAAGGA
GGAACCGACTTGTTCTAAAACGCTTATGCCGGTATTGTTGTTTTGGGATTTTTGAATGTTTGCCTTATTGGTGGCGTTATCTTTCTCTCTTA
CAAATTATTCTTTTCTAAAGGAAATAAAGAAGGAAAGAAGGAAGAAGATAAAAAGAAGGAAGGGGAAGGAATAAGAAAGAAGGGGA
AGAACCTAAAAAGTAA
>MjEFF12c1
ATGTGTTCTGTTTCTCTGTTTGTTGGGAGTTTGCTTCGTTTGACATTTTTGGTGTTTATTGTTTTTGGATTTGTGGAGGGGAATGAGGAACC
GACTTGTTCAAAAACGCTTATGCCGGTATATGTTGTTTGGGAATTCTGAATGTTTGCCTTATTGGTGGCACTTGTTCAAAAACGCTTATGC
CGTTATATGTTGTTTTGGGACTTTTGAATGTTTGCCTTATTGGTGGCGTTATCTTTCTCTCTTACAAATTATCTTTTCTAAAGGAAATAAAGA
AGGAAAGAAGGAAGAAGATAAAGGAGATAAAAAGAAGGAAGGGGAAGGAGATAAGAAAGAGGGGAAGAACCTAAAAAGTAAAGGA
ATAAAATGTGTAGAAGATGTATTGGGATAAAATGTCGTGTTAATTTTGTGTTATTTTTTTTTGTTTAG
>MjEFF12c2
ATGTTTTCTGTTTCTCTGTTTGTTGGGAGTTTGCTTCGTTTGACATTTTTGGTGTTTATTGTTTTTGGATTTGTGGAGGGGAATGAGGAACC
GACTTGTTCAAAAACGCTTATGCCGGTATATGTTGTTTGGGAATTTTGAATGTTTGTCTTATTGGTGGCGTTATCTTTCTCTCTTATAAATT
ATTCTTTTCTAAAGGAAATAAAGAAGGGAAGAAGGAAGAAGATAAAAAGAAGGAAGGGGAAGGAGATAAGAAGAAGGGGAAGAACC
TAAAAAGTAA
>MfEFF12a
ATGCCGGTATATGTTGTTTTGGGAATTTTGAATGTTTGTCTTATTGGTGGCGTTATCTTTCTCTCTTACAAATTATTCTTTTCTAAAGGAAAT
AAAGAAGGAAAGAAGGAAGAAGATAAAGGAGATAAAAGAAGGAAGGGGAGGGAGATAAGAAAGAAGGGGAAGAACCTAAAAAAGA
AGATGTGATTGGGATAAAATGTCGTTGTTGTCTTTTCGCTAAATACAATCAAAAGAACCAGAGATTGTTCATTCTAGTATTAGGGGAGCA
GGCCAAGGGTACCCAGGGCACCGCAGAACTATAACTTCGCTCAAAATTGTCGAATCCGTGAAGCCCTGA

>MfEFF12b
ATGCCGGTATATGTTGTTTTGGGAATTTTGAATGTTTGCCTTATTGGTGGTGTTATCTTTCTCTCTTATAAATTATTCTTTTCTAAAGGAAAT
AAAGAAGGGAAGAAGGAAGAAGATAAGGGAGATAAAAGAAGGAAGGGGAAGGAGATAAGAAAGAAGGGGAAGAACCTAAAAAGTA
A
>MeEFF12
ATGCCTTCTAGTTCTGTTTCTCTGTTTGTTGGGAGTTTGCTTCGTTTGACATTTTTGGTGTTTATTGTTTTTGGATTTGTGGAGGGGAATGA
GGAACCGACTTGTTCAAAAACGCTAATGCCGGTATTGTTGTTTTGGGACTTTTGAATGTTTGCCTTATTGGTGGCGTTATCTTTCTCTCTTA
CAAATTGTTCTTTTCTAAAGGAAATAAAGAAGGGAAGAAGGAAGAAGATAAAGGAGATAAAAAGAAGGAAGGGAAGGAGATAAGAAA
GAAGGGGAAGAACCTAAAAAGTAA
>MhEFF12
ATGTGTTCCGATTCTGTTTCGTTGTCTATTGGATATTTGTTTCGTTTGATGTGTTTGATGTTTTTTGTTTTTGGATTTGTGAAAGCCACTGAA
CCGACTTGTTCTCAAACACTTTTGCCATTATATATGGTTTGGGATTTTTGAATGTTTGTCTTATTGGTGGAATTATCTTTCTCTCTTTCAAAA
TATTTTCTTCAAAAGGAAATAAGAAAGAAGAAAATAAAAAAGAAGATAAAAAAGAAAATGAAGAACCAAAAAAGTAAAGGAATAA

Figure S2. Nucleotide sequences of *EFF12* encoding genes identified in RKN species.
