# Supplementary material for: The root‐knot nematode effector MiEFF12 targets the host ER quality control system to suppress immune responses and allow parasitism
Source: Mol Plant Pathol. 2024 Jul 4;25(7):e13491. doi: 10.1111/mpp.13491 (PMC11222708; doi:10.1111/mpp.13491)
Supplement: Supplementary file 3 — Figure S3. Ectopic expression of MiEFF12a in Arabidopsis thaliana does not affect root development. [file MPP-25-e13491-s002.pdf]

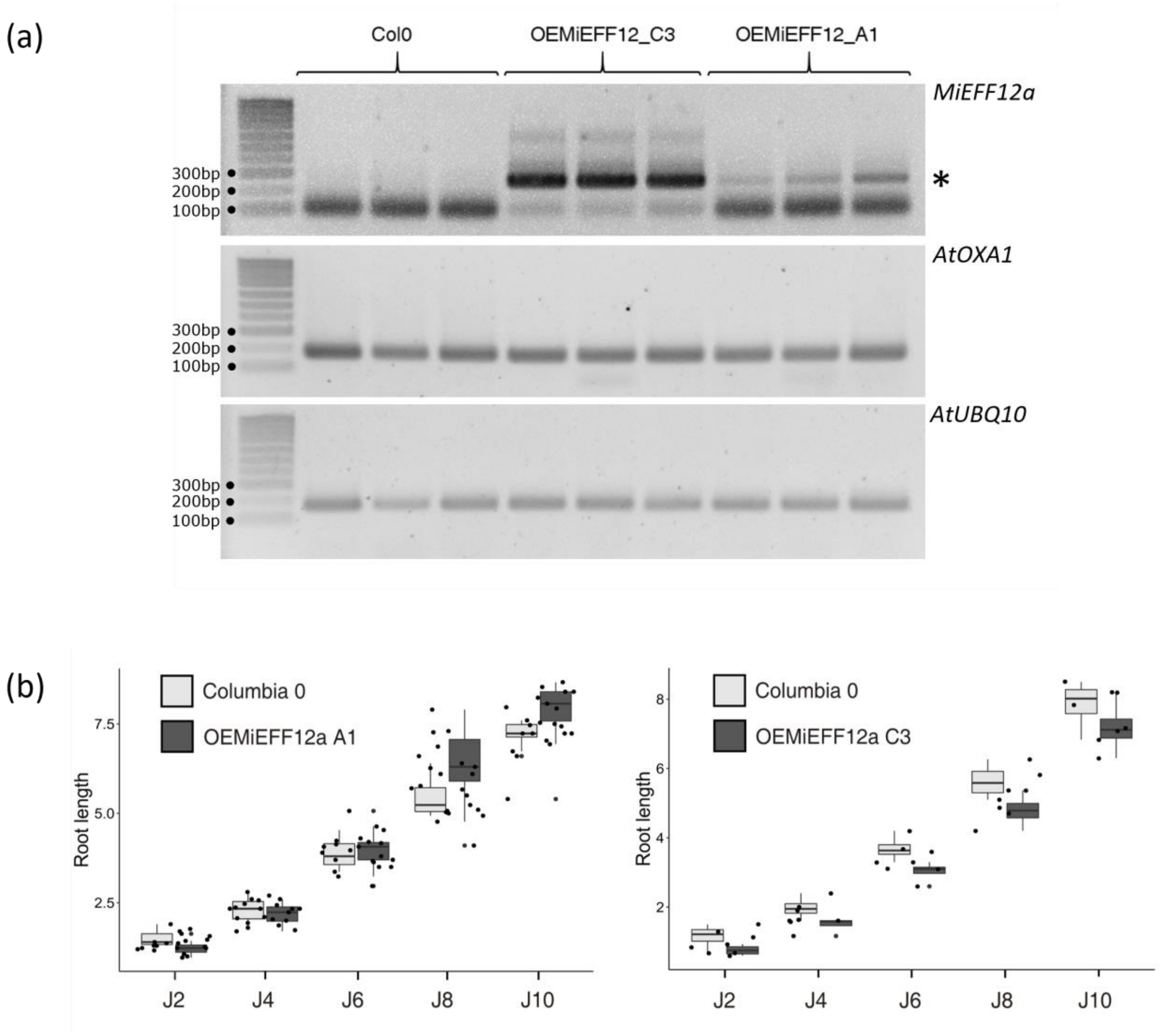

Figure S3. Ectopic expression of *MiEFF12a* in *Arabidopsis thaliana* does not affect root development. Two independent *Arabidopsis* transgenic lines expressing *MiEFF12a* without the SP under the control of the P35S promoter were selected, and homozygous T3 was used for further analysis. (a) Semi-quantitative RT-PCR analysis of *MiEFF12a* expression in the two independent line (#C3 and #A1) was performed with specific primers from the RNA isolated from the *Arabidopsis* seedlings. The *AtOXA1* and *AtUBQ10* genes were used as internal loading control. (b) Root length (in cm) was measured in the transgenic lines every two days from days 2 to 10 after the transplantation of seven-day-old plantlets on phytagel medium. No significant difference was observed between the wild type and any of the *MiEFF12*-overexpressing lines (#A1 and #C3).
