# Supplementary material for: The root‐knot nematode effector MiEFF12 targets the host ER quality control system to suppress immune responses and allow parasitism
Source: Mol Plant Pathol. 2024 Jul 4;25(7):e13491. doi: 10.1111/mpp.13491 (PMC11222708; doi:10.1111/mpp.13491)
Supplement: Supplementary file 4 — Figure S4. MiEFF12a interacts with SlPBL1, SlPBL2 and SlBZIP60 in yeast. [file MPP-25-e13491-s017.pdf]

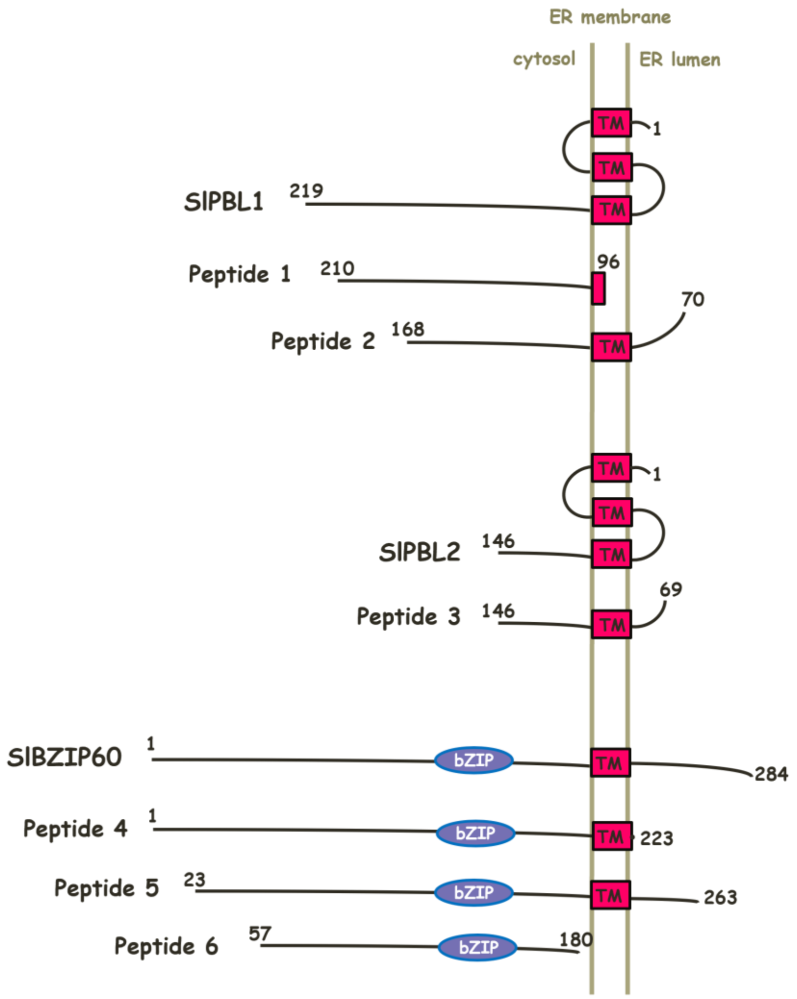

Figure S4. MiEFF12a interacts with SlPBL1, SlPBL2 and SlBZIP60 in yeast. Six different peptides, matching SlPBL1, SlPBL2 or SlBZIP60, were captured when performing a Y2H screen with MiEFF12a as a bait. Two peptides, peptide 1 (amino acids 96 to 201) and peptide 2 (aa 70 to 168), corresponding to SlPBL1, were captured twice and nine times, respectively. One peptide, peptide 3 (amino acids 69 to 146), corresponding to SlPBL2, was captured once. Three peptides, peptide 4 (amino acids 1 to 223), peptide 3 (aa 23 to 263) and peptide 6 (aa 57 to 180), corresponding to SlBZIP60, were captured once, five times and once, respectively.
