# Supplementary material for: The root‐knot nematode effector MiEFF12 targets the host ER quality control system to suppress immune responses and allow parasitism
Source: Mol Plant Pathol. 2024 Jul 4;25(7):e13491. doi: 10.1111/mpp.13491 (PMC11222708; doi:10.1111/mpp.13491)
Supplement: Supplementary file 5 — Figure S5. Nucleotide sequences of SlPBL1 and SlBZIP60. [file MPP-25-e13491-s013.pdf]

>SlPBL1/Solyc12g005910
ATGTTACCTCAAGTGCAGCTTTTGTTCATGGTGGTGTTCTTGGAAATGGGGTTGATTTTG
ATGTTTCTGTTCAGAACCCCTTTGAGGAAACTGATAATTATGAGTCTTGATCGTGTTAAA
AGAGGCAGTGGACCGTTGATTGTTAAATCTGTAGCAGCTACTGTTCTTGTGATCATGATT
TATACTGTTTATTCTATTAAGGAATTGCAGTCTCGGCCTGCTGATTCTGTGAATCCTACC
GATCAAATCCTTCTTGCTCATCAGATTCTTGAAGCTGCTCTTATGGGATTCTCTCTATTC
CTCGGACTGATGATTGACAGGCTACACCATTACATAAGGGAGCTTCGATTACTAAGGAAG
ACCATGGAGGCTGTGAAGAAGCAGGATCGTACATTGGACAACATCAAGAACGGCGAGGCC
AGTACCTTAAGGGATGAAATCTCCTCCTTGAGGAACAGGATGAAGCAGCTGGAATCAGAA
TCGGAGGCAAAAGAAAAGGAGGTGCAATCTCAGAGAGCAAATTCTGATTCTCTCAAGGGT
CAATCTGAAAAATTGCTGCTTGAATACGATAGGATCCTGGAAGAGAATCAGAATCTTCGA
AGCCAATTGCAATCAGTTGACGATACCCTGTCACATTCTGATAACAAGAAGAACACATAG

>SlPBL1/Solyc12g005910
MLPQVQLLFMVVFLEMGLILMFLFRTPLRKLIIMSLDRVKRGSGPLIVKSVAATVLVIMI
YTVYSIKELQSRPADSVNPTDQILLAHQILEAALMGFSLFLGLMIDRLHHYIRELRLLRK
TMEAVKKQDRTLDNIKNGEASTLRDEISSLRNRMKQLESESEAKEKEVQSQRANSDSLKG
QSEKLLLEYDRILEENQNLRSQLQSVDDTLSHSDNKKNT

>SlBZIP60/Solyc04g082890
ATGATCGATAACATCGATGATATCATCAACTGGGACGATGTAGATCACATCTTCCACAAC
GTTCTAGACAATCCCGACGATGATCAATTCACTCTTCATGATTCCTCCCCACAGTCATTC
CAGCAGATCGAGCAGCTTCTTATGAACGATGACGATTTCGGTCTTGTCTCTGATCCTCAG
TTTGCTGCCGAATCTCTTTCTGACTTCCTCGTCGATTCTCCTCTTCACTCCGATCATTCT
CACTCTCCTGCTGAACAAGCCATTGGATTCTCCGATCCCAAGGTTTCAAGTGCCGATCAG
GACAAACACAAGGTTTCCCAGTCGCCTTCTGACGGCGACGACGAACTAAACAACCATGAC
CCCGTCGATAAGAAGCGCAAGAGGCAATTGAGGAATAGAGATGCAGCTGTGAGGTCACGA
GAGAGGAAGAAGTTGTACGTTAGGGATCTTGAGTTGAAGAGTAGATACTTTGAATCAGAA
TGCAAAAGGCTGGGGTTTGTTCTCCAGTGCTGTCTTGCTGAAAATCAAGCTTTACGCTTT
TCTTTGCATAATAGCAGTGCTAATGGTGTTTCTATGACCAAGCAGGAGTCTGCCGTGCTC
TTTTTGGAATCCCTGCTGTTGGGTTCCCTGCTTTGGTTCCTGGGCATCACATGCCTGCTC
ATTCTACCCAGCCAAACCTGGTCAGTTCCAGAAGAAAGTCAGGGAAGCAGAAACCACGGG
CTTCTGGTTCCAATAAAGGAGGGAAAAAAGACTAGTCGGATTTTGTTGTTCCTGTCCTTC
ATGATGAGCAAAAGATGCAAAGCTTCAAGATCGAGGATGAAGCTCAACCCCCATCCCTTG
GGAGTTGTGATGTGA

>SlBZIP60/Solyc04g082890
MIDNIDDIINWDDVDHIFHNVLDNPDDDQFTLHDSSPQSFQQIEQLLMNDDDFGLVSDPQ
FAAESLSDFLVDSPLHSDHSHSPAEQAIGFSDPKVSSADQDKHKVSQSPSDGDDELNNHD
PVDKKRKRQLRNRDAAVRSRERKKLYVRDLELKSRYFESECKRLGFVLQCCLAENQALRF
SLHNSSANGVSMTKQESAVLFLESLLLGSLLWFLGITCLLILPSQTWSVPEESQGSRNHG
LLVPIKEGKKTSRILLFLSFMMSKRCKASRSRMKLNPHPLGVVM

Figure S5. Nucleotide and amino acid sequences of SlPBL1 and SlBZIP60
