# Supplementary material for: The root‐knot nematode effector MiEFF12 targets the host ER quality control system to suppress immune responses and allow parasitism
Source: Mol Plant Pathol. 2024 Jul 4;25(7):e13491. doi: 10.1111/mpp.13491 (PMC11222708; doi:10.1111/mpp.13491)
Supplement: Supplementary file 6 — Figure S6. MiEFF12a and SlPBL1 colocalize in Nicotiana benthamiana epidermal leaf cells and both MiEFF12a‐ and SlPBL1‐GFP fusions were localized in large subcellular structures mostly juxtanuclear. [file MPP-25-e13491-s018.pdf]

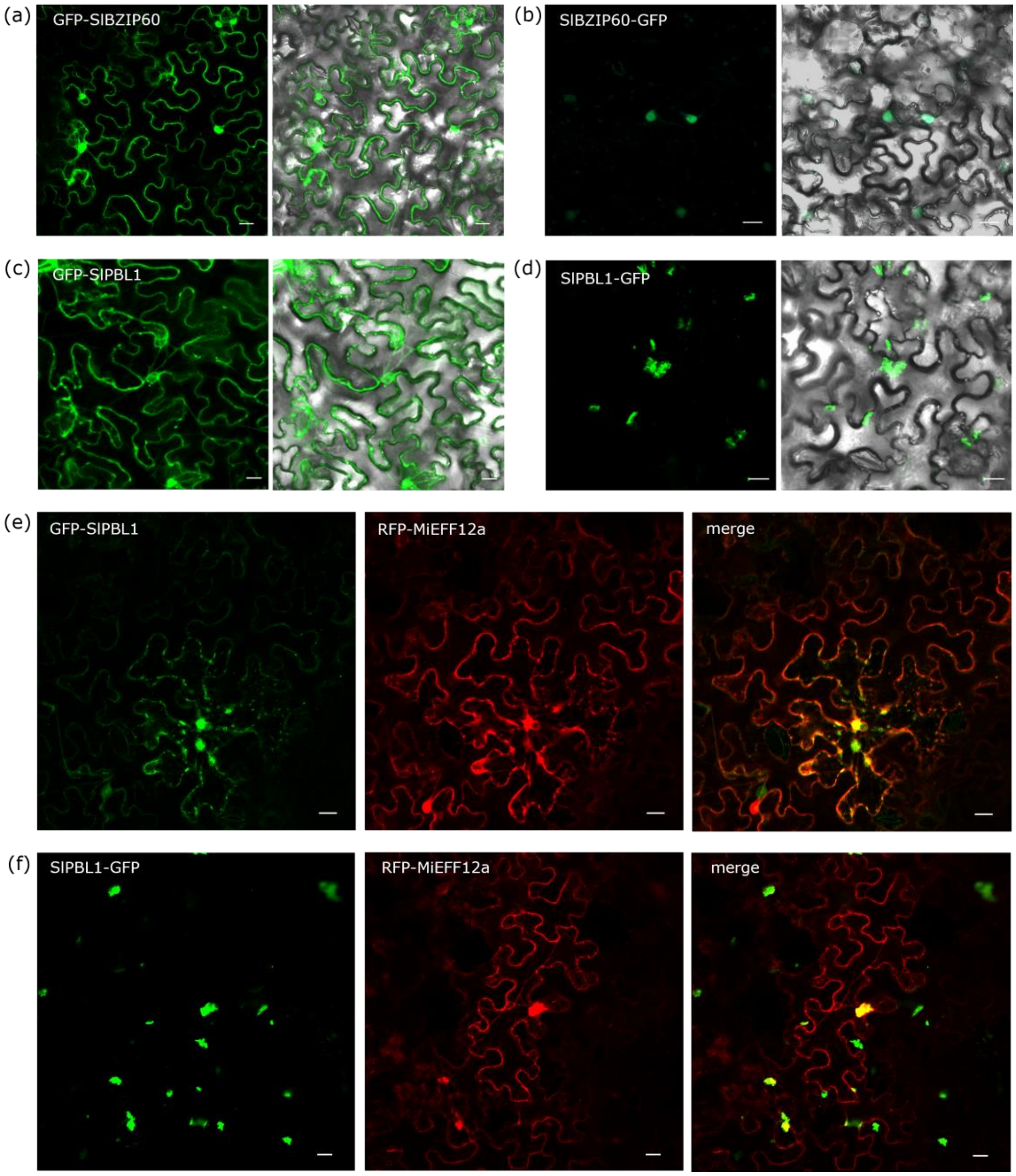

Figure S6. MiEFF12a and SlPBL1 colocalize in *N. benthamiana* epidermal leaf cells and both MiEFF12a- and SlPBL1-GFP fusions were localized in large subcellular structures mostly juxtanuclear. (a-b) Single-plane confocal images of *N. benthamiana* leaf cells infiltrated with *Agrobacterium tumefaciens* and producing SlBZIP60lPBL1, fused to the C-terminal end (a) or to the N-terminal end (b) of the green fluorescent protein (GFP) reporter (GFP-SlBZIP60 and SlBZIP60-GFP; green signal; left pictures). Overlays of differential interference contrast and fluorescence images are shown (right pictures). (c-d) Single-plane confocal images of *N. benthamiana* leaf cells infiltrated with *Agrobacterium tumefaciens* and producing SlPBL1, fused to the C-terminal end (c) or to the N-terminal end (d) of the GFP (GFP-SlPBL1 and SlBZIP60-GFP; green signal; left pictures). Overlays of differential interference contrast and fluorescence images are shown (right pictures). (e-f) Single-plane confocal images of *N. benthamiana* leaf cells infiltrated with *A. tumefaciens* and producing SlPBL1, fused to the C-terminal end (e) or to the N-terminal end (f) of the GFP (GFP-SlPBL1 or SlPBL1-GFP; green signal) and MiEFF12a fused to the C-terminal end of the red fluorescent protein (RFP) (RFP-MiEFF12a: red signal). Overlays of fluorescence images are shown (merge). Scale bars: 20 µm.
