# Supplementary material for: The root‐knot nematode effector MiEFF12 targets the host ER quality control system to suppress immune responses and allow parasitism
Source: Mol Plant Pathol. 2024 Jul 4;25(7):e13491. doi: 10.1111/mpp.13491 (PMC11222708; doi:10.1111/mpp.13491)
Supplement: Supplementary file 7 — Figure S7. Nucleotide sequences of Nicotiana benthamiana BZIP60 and design of the virus‐induced gene silencing (VIGS) construct. [file MPP-25-e13491-s008.pdf]

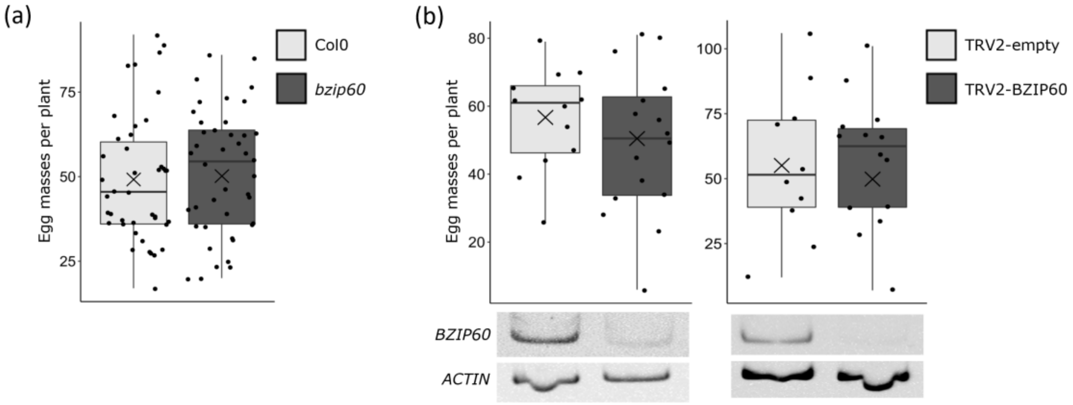

Figure S7. Plant BZIP60 is not required for *M. incognita* parasitism. (a) Box-and-whisker plots of egg masses per plant in Col0 control line and *bzip60* mutant line six weeks post infection with 200 *Meloidogyne incognita* second-stage juveniles (J2s). Box indicates interquartile range (25th to the 75th percentile). The central line within the box represents mean value. The cross represents average value (n=40 plants per line). Whiskers indicate the minimum and maximum values for the normal values present in the dataset. Statistical significance was assessed in Student's t tests. No significant difference was observed between the wild-type and the *bzip60* mutant. (b) Infection test on *N. benthamiana* control plants (TRV-empty) and plants in which *NbBZIP60* was silenced (TRV2-BZIP60). The efficiency of *NbBZIP60* silencing was monitored by RT-PCR 10 days following TRV inoculation. The *ACTIN* housekeeping gene was used for data normalization. Galls were counted six weeks after inoculation with 200 M. incognita second-stage juveniles (J2s) per plant. Results from two independent experiments are shown (n=10 to 16 plants per condition). The cross represents average value. Box indicates interquartile range (25th to the 75th percentile). The central line within the box represents mean value. Whiskers indicate the minimum and maximum values for the normal values present in the dataset. Statistical significance was determined in Student's t test and no significant difference was observed between controls and TRV-BZIP60 plants.
