# Supplementary material for: The root‐knot nematode effector MiEFF12 targets the host ER quality control system to suppress immune responses and allow parasitism
Source: Mol Plant Pathol. 2024 Jul 4;25(7):e13491. doi: 10.1111/mpp.13491 (PMC11222708; doi:10.1111/mpp.13491)
Supplement: Supplementary file 8 — Figure S8. Plant BZIP60 is not required for Meloidogyne incognita parasitism. [file MPP-25-e13491-s006.pdf]

>NbBZIP60/Niben101Scf24096g00018
tttccttgctatttcctcggttaccttctctttggagtttggacccttaacctctaagaaatacggcgtgtatca
cgtgactatttattggccccatccacaggtcccaaaatcgtagctgtagctgtagttcccccaccccaaaccccg
tatttcttgttgaattttggttcaatcatcccaatctgggttgtagaataggcg**ATG**GTGGATGACATCGATGAT
ATCGTTGGACACATCAATTGGGACGATGTAGATGACCTCTTCCACAACATTCTAGAGGATCCCGCCGACAATCTC
TTCTCTGCTCATGATCCGTCCGCGCCGTCTATCCAGGAGATCGAGCAGCTTCTCATGAACGATGATGAAATCGTC
GGTCACGTGGCTGTCGGAGAGCCTGATTTTCAACTTGCTGACGACTTTCTCTCCGACGTGCTAGCCGATTCTCCT
GTTCAGTCCGATCTTTCTCACTCTGATAAAGTCATTGGATTCCCCGATTCCAAGGTTTCAAGTTGCTCAGAGGTT
GATGATGACGACAAAGACAAGGAGAAGGTTTCCCAGTCGCGGATTGACTCTAAGGACGGCTCTGACGAACTAAAC
TGTGATGATCCCGTCGATAAAAAGCGTAAGAGGCAATTGAGAAACAGAGATGCAGCTGTCAGGTCACGAGAGCGG
AAGAAGTTGTATGTTAGGGATCTTGAGTTGAAGAGTAGATACTTTGAATCAGAGTGCAAGAGGTTGGGGTTAGTT
CTCCAGTGCTGTCTTGCAGAAAATCAAGCTTTGCGCTTCTCTTTGCAGAATGGCAATGCTAATGGTGCTTGTATG
ACCAAGCAGGAGTCTGCTGTGCTCTTGTTGGAATCCCTGCTGTTGGGTTCCCTGCTTTGGTTCCTGGGCATCATA
TGCCTGCTCATTCTTCCCAGCCAACCCTGGTTAATTCCAGAAGAAAATCAACGAAGCAGAAACCACGGTCTTCTG
GTTCCGATAAAGGGAGGAAATAAGGCTGGTCGGATTTTTGAGTTCCTGTCCTTCATGATGGGCAAGAGATGCAAA
GCTTCAAGATCGAGGATGAAGTTCAATCCCCATTCTTTGGGAATTGTTATG**TGA**ctcattgtgatcaaatccttt
cccttggtggatcctgcttcctttgagttctttaatttgtatcataggctttctgtatgagtctttgcttttaca
tctgaaggacgccgttcagtgttttagttaatgacgtatgagcttactaagatctatgcaacttttttaacagttc
cagcctaaatttgtttcgtctctgttttgcattttcttttcttcattctttctgcccgagtggttgttgtggcat
ggtagcaggattacaaggccaccacaagatgctacaaaattttcaaaattatgaaatcgatgtgaagatgaatgg
ttagatttctattaagtttgatatataacgatttagcatcttcaagtatgttctcagtttgatggtgaattgagg
ctgtatgtgttttccctctttttttctactctataatggcttggaacggaaaaattggatagtctggaagtgtat
tgacaaatgagaaaagaagcatctccgcaaatgtatgcactccttttcttctttggtatgggtaaa

Figure S8. Nucleotide sequences of Nicotiana benthamiana BZIP60 and design of the VIGS construct. The mRNA sequence of NbBZIP60 is shown. The open reading frame (ORF) appears in upper case and the untranslated regions (UTR) in lower case. The sequence selected by the VIGS tool to target NbBZIP60 is highlighted. Primers used to validate NbBZIP60 silencing by RT-PCR are highlighted in blue.
