# Supplementary material for: The root‐knot nematode effector MiEFF12 targets the host ER quality control system to suppress immune responses and allow parasitism
Source: Mol Plant Pathol. 2024 Jul 4;25(7):e13491. doi: 10.1111/mpp.13491 (PMC11222708; doi:10.1111/mpp.13491)
Supplement: Supplementary file 9 — Figure S9. Nucleotide sequence of Nicotiana benthamiana PBL genes targeted by the virus‐induced gene silencing (VIGS) approach. [file MPP-25-e13491-s011.pdf]

>NbPBL1a
CCAAAGCAATGGGAAACGAAAAAAAAAAATGGAAGACAGATCGCTGCTTCAGAAAATAAGAAAATATATTTTTCC
TATATTTAAGCTCTTGCCGACGCTGGTTTTTGGCGGCGTAAGCCGCGTGCCAGAAAAACAAGTGTATCATTTAAG
ACAAGTCCACCTCTACTCTCTTCTCAAATATCAACCCCACTTTCTGCACTTTCCACAAATAGCAAATTTCTCAAT
CCAAACTCTCCTTACCCAGTAGAAGAAAGAGAAAACTTTGCTGAATTTCAGTTCCCCCACAAAAAGAGCAGTTAA
TTTGGTACTAATATAAAAAAAAATGTTAGCACAAGTACAGCTTCTGTTTATTGTGGTATTCTTAGAAATGTCACT
AATTTTGTTGTTTCTCTTCAAAACCCCTTTGAGGAAACTAATAATCATGACTCTTGACCGTGTCAAACGAGGTCG
TGGACCGTTAATTGTTAAATCTGTAGCTGCTACTGTTCTTGTGATCATGATTTACACTGTTTACTCTATTAGGGA
ATTGCAGTCTCGCCCTACTGATACTGTTAATCCCACTGATCAAATCCTTCTTGCTCATCAGATTCTCCAAGCTGC
TCTTATGGGATTTTGTCTATTCCTTGGACTGATGGTAGACAGGCTACACCATTACATAAGGGAGCTTCGCTTACT
CAGGAAGACGATGGAAGCTGTAAAGAAGCAGGATCGCGCATTGGACAATGGCAAGAATGGTGAAGCCAGTAACCT
AAAGGATGAAATCTCCTCCTTGAGGAACAAGGTAAGGCAGCTGGAATCCGAAAACGAGGCAAAAGAAAAGGAGGT
AAAATCTCAAAAGGCTAATTCAGATTCTCTCAAGGGTCAATCTGAAAAATTGCTGCTTGAATACGACCACTTGCT
GGAACAAAACCAGAATCTTCGAAGCCAATTACAATCAGTTGACCAAAATGTATCACATTCTGATAGCAAAAAGAA
GACCTAGAATGGCATACATCTGTTTTCGGGCCATCAACTGTAACAGTTACCATCCTGAGAATTTTGGTTAAAGAA
GTGTGTTGTGTAGCTTGTCTTTGTCATCATGATAGATTCTTGTAAAATGGTAGTAATATATTGTAGTGGCTCCAA
ACCCTGGATTATCATAAACCACTCCTGTTTTCTATCTTTCACTGTGCTTGAAACTATTTTTGTGTTCCCAGTGGG
GGTGATTATACAATAATATTTTGCTCTTGAGGGAGTCTCAGATTTTTTTACCTAGCAAGGGACCCTTTCAGTGAT
GTGGATAGCAGCATTTTCAAATTAAATCGTATGATATCATTTATAACACTTTAAATGTAAATTTAAGAAGTCAGG
TAGGTTCAATTCTTG

>NbPBL1b
CTCTCTATTTGCTCTTGTTCTTGGGTTATTGTTTTGTCTTTTATATTATTTCATAACATTACTATATTTAAAGGT
CTTGCCGACGCTAGTTTTTGCCAACCGAGTCTTGCAGGCGTTAAGGTCTTTGCCGACGCTAGTTTTTGGTGGCGT
AAGCCGCGTGACACAGAAAAATAAGTGTATCATCCACCTTTACACTCTTCCACAATAAAGCCACTCTCTTCTCAA
ATATCAACCCCTTTTTCTGTACTTTCCATATATAGCAAATTTCTCTGCTCAGCTCTCCTTACTCCCCCCAAAAAA
AGAAAGAGGAAACTTTGCTGAATTTCAGTTCCCTACAAAAAGAGCAGTTATTTTGGTACTAATATCAAAAAAATG
TTAGCACAAGTACAGCTTCTGTTTATGGTGGTATTCTTAGAAATGTCACTAATTTTGTTATTTCTCTTCAAAACC
CCTTTGAGGAAACTAATAATCATGACCCTTGACCGAGTCAAACGAGGCCGTGGACCGTTGATTGTTAAATCTGTA
GCTGCTACTGTTCTTGTGATCATGATTTACACTGTTTATTCTATTAGGGAATTGCAGTCTCGCCCTACTGATACT
GTTAATCCCACTGATCAAATCCTTCTTGCTCATCAAATTCTCCAAGCTGCTCTTATGGGATTTTGTCTATTCCTT
GGACTGATGGTAGACAGGCTACATCATTACATAAGAGAGCTTCGTTTACTTAGGAAGACCATGGAAGCTGTAAAG
AAGCAGGATCGCGCACCGGATAATGGCAAGAATGGTGAAGCCAGTAACCTAAAGGATGAAATCTCCTCCTTGAGG
AACAAAGTAAGGCGACTGGAATCCGAAAACGAGGCAAAAGAGAAGGAGGTAAATTCTCAGAAGGCTAATTCAGAT
TCTCTCATGAGTCAATCTGAAAAATTGCTGCTTGAATACGACCGGTTGGTGGAAGAAAATCAGAATCTTCGAAGC
CAATTGCAATCAGTTGACCAAAATGTATCACATTCCGATAGCAAAAAGAACACCTAGAATATCGTACATCTGTTT
TCTGGCCATCAACTGTAGTAGTTACTATCTTGAGAATTTTGGTTAAAGAAGTGTGTTGTGTAGCTTGTCTCTGTC
ATCATGATAGATTCTTGTAAAATGGTAGTAATATATTGTATTGGCTCCTTACCCTGGATTATCCTAAACCACTCC
TGTTTTCTGTTTTTTACTGTGCTTGAAACTATTTTTGTGTTCCCAGTGAGGGTGATATACAACAATATTTTGCTC
TTGAGGGACTCTCAGATCTTTTTATTTCGCAAGGGACCATTTCAGTGATGTGGATAGAAACATTTTCAAATTATA
CCGTGTGATATCATTTATAACACTTTAAAAGTAAATTTAAGAAGTCAGGTAGGTTCAATTCTTG

>NbPBL2a
CATTTTCCTACTTCTTAGTCCCAAAGCATATTCCTTTGTTACTAACACGCTTTTAGAGTTGAGTTAAATATTTTC
CTTTACCTAACTAATAAGTCATCACAACAACAACAACAACCAAAAGTTGTGTTTTTTGAACATTCACCGACAATA
TGATTCAACCGTTATTCGCACTTGTATTTTTTGAAGTGGCAGTGGTATTGATCCTCGTATTTCGAACCCCTTTAA
GAAAACCTGTTTTGATGGCATTGGATAAATCAAAACAAGGGAGGGGACCTGTCATAGTAAAGAGTGCTGGTGGAA
CCTTATTTGTGGTTTTAGTCTCAATCTTGTTCAATGTCACTATTATTCAGAACCGTGCCACGGAATCTGGCACTG
TCAATCCAACTGATCAAGTTCTATTGGCTAATCACCTCCTAGAAGTGTCACTATTGGGATTTTCATTGTTTCTTG
CATTGGTCATAGATAGACTTCACTACTACATAAAAGAGCTGCGTCTGCTTAGGAAGACTTTGGAGGCAGAAAAGA
AAACAAAACAGAGCCGCGAGGTGGAAAATGTGACACCAAAAAGTCCGACCAAACATACATAGAAATATGGAGGCT
ATACATCTAAGTTTTACCAACTATATTTTCTGTTTATGTGATCTTTTAGGTCATTTTTAACACGTATATTTGACA
TTTCCTGGTGTACGACGATGTTTTTCTTTTCTCCAATTATTTTAAAACTCCTTTGTATTCCTAGATGTCTGATGG
TGATTTGTAATTTTTTTATTTTAAGAGCAAACTGTTATTTAACTGCCACTCTTGTAATCAAACTACCAGGAAATA
GAAATGGAAAGGAACCATTAAGTTTCAATGACTTGTCTGTTTGAAAATTTTCAAAAAATAGAGCATGTAAAAAGT
TTTGACAGTTTGCAAACTATTTATTGGAGT

> NbPBL2b
TTTCTTACTTGTTAGTCCCTAAGCATTTTCTTTTGTTAGTAGCACGCTTTTAGAGTAGAGTTAAATATTTTCCTT
TACCTCACTAATAATTCATCACAACAACAACAACTAAAAGTTGTGTTTTTTAAACATTCAACGATAATATGATTC

AACCGTTATTCGCACTTGTATTTTTTGAAGTAGCTGTGGTATTGATCCTCGTATTCCGAACCCCTTTAAGAAAAC
CTGTTTTAATGGCATTGGATAGATCAAAACAAGGGAGGGGACCTGTGATCGTAAAGAGTGCTGGTGGAACCTTAT
TTCTGGTTTTAGTCTCAATCTTGTTCAATGTCACTATAATTCAGAACCGTGCAACGGAGTCTGGCACTGTCAATC
CAACTGATCAAGTTCTATTGGCTAATCACCTCCTAGAAGCGTCATTATTGGGATTTTCATTGTTTCTTGCATTGG
TCATAGATAGACTTCACTACTACATAAAAGAGCTGCGTCTGCTAAGGAAGACTTTGGAGGCAGAAAAGAAAACAA
AACAGAGCCGCGAGGTGGAAAATGTGACACCAAAAAGTCCGACCAAAAGTACATAGAAATATGGAGGCTATATTT
CCTGTTTGTTTGATCTTTTAGGTCGTCTTTAACACGTATATTTGGCATTTCTTGGTGCATGATGATGTGGTTTTT
TTTTCTCCAATTATTTTAAAACTCCTTTATATTCCTAAATGTCTGATGATGATTTGTAATTTGTTTATCTAAGAG
TATACTGTTATTTAACTGCCACTCTTTTTATCAAATTACCAAGAAATATAAACGGAAAGGAACCATAAAGTTTCA
AGGACTTGTTTGTTTGAAAATTTTCAAAAAATAGAGCATGCAAAAAGTCTTGAAAGTTTGCAAACTCTATTTATT
GGAGT

>NbPBL3a
AAATTAAATTCTTTTTTCTCTTGGTGCTCATGCACTAACCGCTCACCAACTGCTCACACCCGACGTATTTTCTCT
CGATCTGGAAAACAAAAGAACAAAACAAAATCCAGGAGAATGATCCAAGTGTTATTCAGTGTGATATTCGCCGAA
ATGGCGTTAATCGTGATGTTCGTATTCAAGACGCCATTGAGGAAGCTGGTGATAATGGGCCTGGACCGTATTAAG
AGAGGCCGCGGCCCAATTGTCGTCAAAACCATCGGCGGAACCATCTTCGTGGTCATGCTGTCAACAATCTACAGT
GTGGTGTCGATCCGCAAACGCTGGATCGATGAAGATGGCAATATTACTCCTACTGATCAGATTCTTATGGCTCAA
CATCTTCTAGAAGCTTCCCTCATGGGTTTTTCCCTATTCCTTGCTCTGATGGTTGACAGACTGCACCACTATATA
AGAGAACTTCGTATGAGGAGGAAGAGCATGGAAGCTGTGAATAAGCAAAATAGAGGCTTTGAGGATGGGAAGAAT
GGCGCTTCTGACGATATGAAGTCTTTGGAGGAAGAGGCAAGCGCATTGCGTTTGAAAATAAAGAAGCTGGAGTTA
TGTCTGGAAGAAAAGGCTAGAGAAACAAGCGATGCAGAAGCCAATGCAGTTGCTCTTAAAAAGCAAATCAGAAAA
CTTTCATCTTGAAATGATTCCTTACTTGAGGATAACCAACCTCAATTGCAAGCACTGGATCAGAGATTGTCATCT
GATAGCAAAGAGGATTGATATGGAATCTCGTCTCACATGTCGTCCCTTACTCATCATTACGAAGTTCTGTTATGA
TTTTCTTTTAATAGCTTAGTGCCAATCATGCTCAAAATATGGACACTGACTGTAGAAAATTTCTAAGTTACCATA
GTTTTTTGTGATTTCACCCTGGACTAGGCTCTATGGCGGTCTTCAAACATTTCTCCCAGCGAGTTGCATTGCTGG
ATGGAGTCTTTCTTCATTTAGTTTACCAATCCTTCTGAAAGTACTTGTCTATATGTGCTGTAGTTGCCAAATTCA
TCTGTCTCTCCTAAAGGTAC

>NbPBL3b
CTACTATGCTGATTGTGTAACATGGGAATATATTTTTCCTTTTTTGTCTTAAATGAAGTCAGTATTTATTTGCTC
GATTCGAACCAAAATTAATTTCTTTTCTCTCTTGGTGCTCATGCACTAAACGCTCACCAACTGCTCGCACCCGAC
GTATTTGCTCTCGATCTGAAAAAGAAAAGAACAAAACAAAATCCAGGAGAATGATCCAAGTGTTATTCAGTGTGA
TATTCGCCGAAATGGCGTTAATCGTGATGTTCGTATTCAAGACGCCATTGAGGAAGCTGGTGATAATGGGCCTGG
ACCGTATTAAGAGAGGCCGTGGCCCAATTATCGTGAAAACCATCGGCGGAACCATCTTCATGGTCATGCTGTCAA
CAATCTACAGTGTGGTGTCGATCCGCAAACGCTGGATCGATGAAGATGGCAATATTACTCCTACTGATCAGATTC
TTATGGCTCAACATCTTCTAGAAGCTTCTCTCATGGGTTTTTCCCTATTCCTTGCTCTGATGGTTGACAGACTGC
ACCACTATATAAGAGAACTTCGTATGAGGAGAAAGAGCACGGAAGCTGTGAATAAGCAAAACAGAGGCTTTGAGG
ATGGGAAGAATGGAGCGTCTGACGATATGAAGTATTTGGAGGAAGAGGCAAGTGCATTGCGTTTGAAAATAAAGA
AGCTGGAGTTATGTCTGGAGGAAAAGGCTCGAGAAAAAAGCGATGCAGAATCCAATTCACTTGCTCTTAGAAAGC
AATCAGAAAACTTTCGTCTTGAAAATGATTCCTTACTTAAGGATAACCAGAATCTCCACGCTCAGTTGCAAGCAC
TGGATCAGAGATTGTCATCTGATGCCAAAAAGGATTCATGAGGAATGTCGTCCATTACTTGTCATTACGAAGTTC
TGTTACGATTTTCTTTCAATAGCTTAGTGTCATTCGTGCTCAAAATATGGACACCAGAGTAAGAAATTCTAATTA
CCATAAATTTTTTGTTATGTCACTCCACACTAGGCTCTATGGAGGTCCTCAAATATTTGTCCTAGCAGCCTTCTG
AAAGTACATCTTGTCTATATATGCTGTTCTTGCCAAATTCATCTGTTAAGATGGCTTTCTGAAAAGTACTTTGTA
TTTTTGATTTTGATATCCTAATTTTACTGTCTTGAATGCAATTGATTTTCAAGGAAATTTTGTGCATTCGGGTTG
CAGTTATTTTTATCTTCTAGCCGTTTTCTTTTATTTCTCTTCACCTAGAAGCATGTCACATGAGCTTAATTTTCA
GAAGACTTTCCATGCTTGCAGACGTTAGTGAAATTCATCTATCTTGTCTTAAAAACAAAAGGAAAAGGGGCTTAA
CATGGCCCGAAGTTGAG

Figure S9. Nucleotide sequence of *Nicotiana benthamiana PBL* mRNA targeted by the VIGS approach. Part of each sequence targeted by the TRV construct is highlighted in yellow.
