# Supplementary material for: The root‐knot nematode effector MiEFF12 targets the host ER quality control system to suppress immune responses and allow parasitism
Source: Mol Plant Pathol. 2024 Jul 4;25(7):e13491. doi: 10.1111/mpp.13491 (PMC11222708; doi:10.1111/mpp.13491)
Supplement: Supplementary file 10 — Figure S10. MiEFF12a physically interacts in planta with NbPBL1a, NbPBL2a and NbPBL3a. [file MPP-25-e13491-s009.pdf]

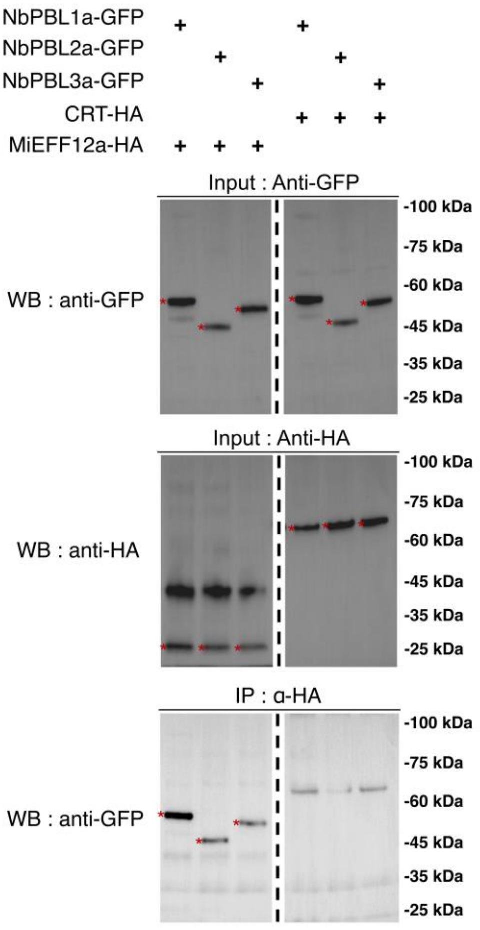

Figure S10. MiEFF12a physically interacts in planta with NbPBL1a, NbPBL2a and NbPBL3a. Co-immunoprecipitation experiments confirmed that MiEFF12a interacted with the full-length NbPBL1a, NbPBL2a and NbPBL3a. NbPBL-GFP fusions were transiently co-expressed with MiEFF12a–HA or MiCRT-HA in tobacco leaves. The Co-IP experiment was performed with anti-HA affinity gel, and the protein isolated was analyzed by immunoblotting with anti-GFP antibodies to detect NbPBLs, and with anti-HA antibodies to detect MiEFF12a and MiCRT.
