# Supplementary material for: The root‐knot nematode effector MiEFF12 targets the host ER quality control system to suppress immune responses and allow parasitism
Source: Mol Plant Pathol. 2024 Jul 4;25(7):e13491. doi: 10.1111/mpp.13491 (PMC11222708; doi:10.1111/mpp.13491)
Supplement: Supplementary file 11 — Figure S11. A chimeric sequence was introduced into the TRV2 RNA to silence the NbPBL genes through virus‐induced gene silencing (VIGS). [file MPP-25-e13491-s003.pdf]

CAGAATTCCTATATTTAAAGGTCTTGCCGACGCTAGTTTTTGCCAACCGAGTCTTGCAGGCGTTAAGGTCTTT
GCCGACGCTAGTTTTTGGTGGCGTAAGCCGCGTGACACAGAAAAATAAGTGTATCATCCACCTTTACACTCTT
CCACAATAAAGCCACTCTCTTCTCAAATATCAACCCCTTTTTCTGTACTTTCCATATATAGCGCCAAAAAGGAT
TCATGAGGAATGTCGTCCATTACTTGTCATTACGAAGTTCTGTTACGATTTTCTTTCAATAGCTTAGTGTCATT
CGTGCTCAAAATATGGACACCAGAGTAAGAAATTCTAATTACCATAAATTTTTTGTTATGTCACTCCACACTA
GGCTCTATGGAGGTCCTCAAATATTTGTCCTAGCAGCCTTCGGATTTTCATTGTTTCTTGCATTGGTCATAGAT
AGACTTCACTACTACATAAAAGAGCTGCGTCTGCTAAGGAAGACTTTGGAGGCAGAAAAGAAAACAAAACA
GAGCCGCGAGGTGGAAAATGTGACACCAAAAAGTCCGACCAAAAGTACATAGAAATATGGAGGCTATATTT
CCTGTTTGTTTGATCTTTTAGGTCGCTCGAGAC

Figure S11. A chimeric sequence was introduced into the TRV2 RNA to silence the NbPBL genes through VIGS. Parts of the sequence in green, blue and purple were selected with the VIGS Tool (https://vigs.solgenomics.net/) for the specific targeting of *NbPBL1a/b*, *NbPBL2a/b* and *NbPBL3a/b*, respectively. The EcoRI and XhoI restriction sites used for cloning into the TRV2 vector are highlighted in blue and green, respectively.
