# Supplementary material for: The root‐knot nematode effector MiEFF12 targets the host ER quality control system to suppress immune responses and allow parasitism
Source: Mol Plant Pathol. 2024 Jul 4;25(7):e13491. doi: 10.1111/mpp.13491 (PMC11222708; doi:10.1111/mpp.13491)
Supplement: Supplementary file 12 — Figure S12. The silencing of PBL genes by virus‐induced gene silencing (VIGS) does not affect Nicotiana benthamiana root development. [file MPP-25-e13491-s019.pdf]

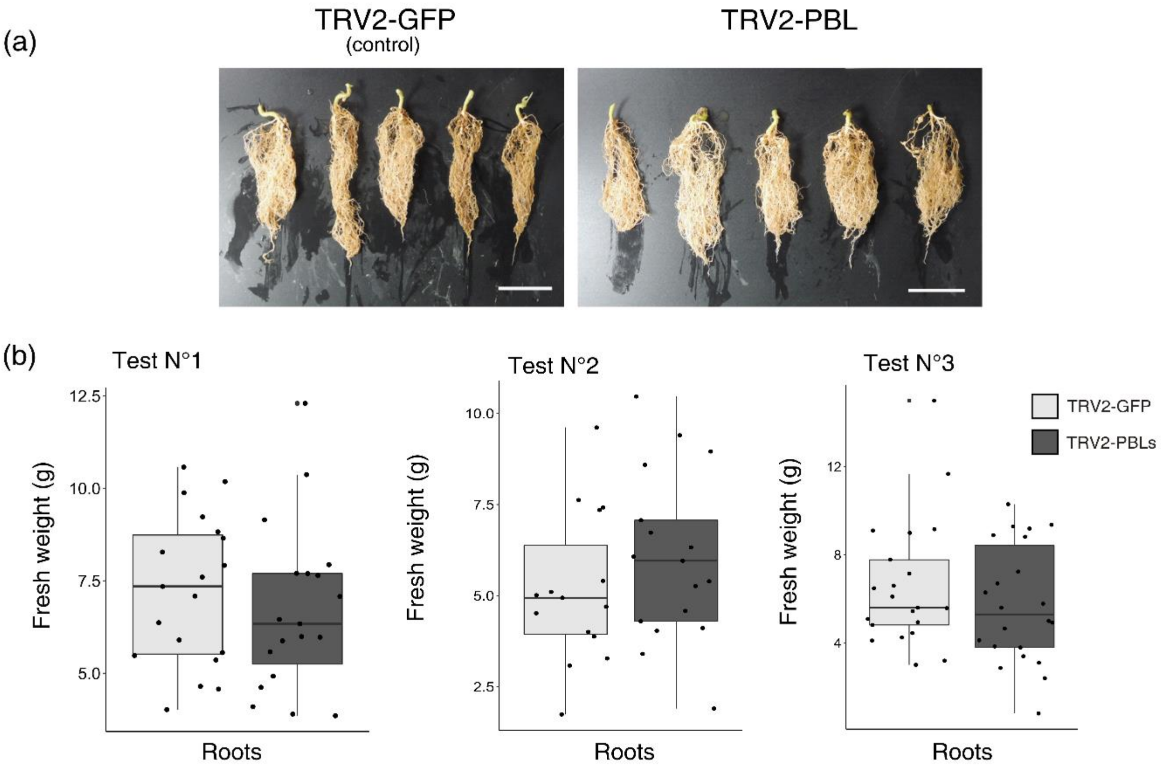

Figure S12. The silencing of PBL genes by VIGS does not affect *N. benthamiana* root development. (a) Pictures showing the root systems of *N. benthamiana* plants with silenced PBL genes (TRV2-PBLs) and control plants (TRV2-GFP). (b) Graph comparing the mass of harvested roots, with no significant difference between TRV2-PBLs plants and controls.
