# Supplementary material for: The root‐knot nematode effector MiEFF12 targets the host ER quality control system to suppress immune responses and allow parasitism
Source: Mol Plant Pathol. 2024 Jul 4;25(7):e13491. doi: 10.1111/mpp.13491 (PMC11222708; doi:10.1111/mpp.13491)
Supplement: Supplementary file 13 — Figure S13. MiEFF12a and SlPBL1a suppress flg22‐mediated reactive oxygen species (ROS) production in Nicotiana benthamiana. [file MPP-25-e13491-s016.pdf]

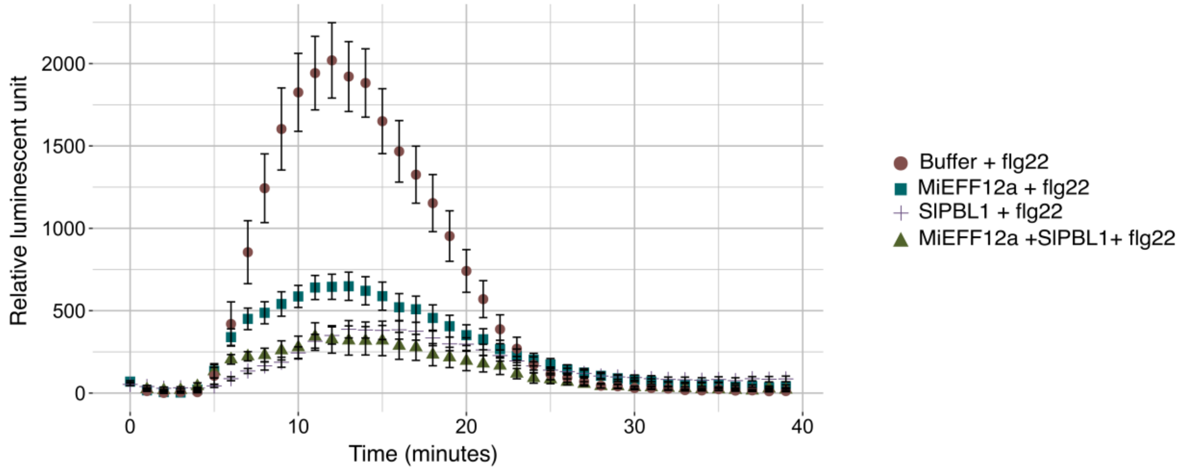

Figure S13. MiEFF12a and SlPBL1a suppress flg22-mediated reactive oxygen species (ROS) production in *Nicotiana benthamiana*. *Agrobacterium tumefaciens* strain GV3101 carrying MiEFF12a or SlPBL1a were used to infiltrate the leaves of *N. benthamiana* plants. Infiltrated leaf discs were collected 48 h post-agroinfiltration and assayed for ROS production in response to treatment with the flg22 elicitor. ROS production was monitored for 40 min, and the values shown are the mean relative luminescence units ± SD for 28 leaf discs. The experiment was repeated three times with similar results.
